# Ultra-fast genome comparison for large-scale genomic experiments

**DOI:** 10.1038/s41598-019-46773-w

**Published:** 2019-07-16

**Authors:** Esteban Pérez-Wohlfeil, Sergio Diaz-del-Pino, Oswaldo Trelles

**Affiliations:** Computer Architecture Department, University of Málaga - Instituto de Investigación Biomédica de Málaga-IBIMA, Málaga, Spain

**Keywords:** Software, Genome informatics, Data processing

## Abstract

In the last decade, a technological shift in the bioinformatics field has occurred: larger genomes can now be sequenced quickly and cost effectively, resulting in the computational need to efficiently compare large and abundant sequences. Furthermore, detecting conserved similarities across large collections of genomes remains a problem. The size of chromosomes, along with the substantial amount of noise and number of repeats found in DNA sequences (particularly in mammals and plants), leads to a scenario where executing and waiting for complete outputs is both time and resource consuming. Filtering steps, manual examination and annotation, very long execution times and a high demand for computational resources represent a few of the many difficulties faced in large genome comparisons. In this work, we provide a method designed for comparisons of considerable amounts of very long sequences that employs a heuristic algorithm capable of separating noise and repeats from conserved fragments in pairwise genomic comparisons. We provide software implementation that computes in linear time using one core as a minimum and a small, constant memory footprint. The method produces both a previsualization of the comparison and a collection of indices to drastically reduce computational complexity when performing exhaustive comparisons. Last, the method scores the comparison to automate classification of sequences and produces a list of detected synteny blocks to enable new evolutionary studies.

## Introduction

Due to technological advances in the past few decades, sequencing whole genomes is becoming cheaper at an exponential rate^1^. As a result, an increasing collection of whole sequenced genomes is becoming publicly available, ranging in size from less than two hundred thousand base pairs^2^ to more than 22 * 10^9^ base pairs^3^. Sequenced genomes are increasing not only in number but also in breadth: larger eukaryotic genomes, such as those of mammals and plants, are being sequenced. These newly incorporated sequences are slowly completing our understanding of organismal evolution, answering questions such as “Which organism arose first?” and “Which evolutionary events caused species divergence?”. However, to improve our understanding of these topics, new data are being included in databases, but doing so comes at a price: the *more genomes* and *larger genomes* trend is aggravating a scenario where it is unthinkable to perform genomic experiments without the aid of extensive computational resources, but furthermore, even current computational methods are being left behind with the increasing complexity.

Currently, most of the handling, exploration and curation of genomic information (such as identifying coding regions or generating pairwise synteny maps) is performed manually and curated with platforms dedicated to this purpose, which often include a repository of precomputed genome comparisons. For instance, NARCISSE^4^, Genomicus^5^ and SynFind^6^ provide such data along with other tools for exploration purposes such as genome browsers and karyotype analysers^7^. Although these resources contain high-quality curated information, they usually rely on previously computed data and typically do not allow users to run new experiments on demand. As a result, when new experiments are supported by these platforms, it is common to employ restricted comparison methodologies such as gene-based approaches (see CoGe^8^) to lower computational demands. However, these approaches are often based on the BLAST^9^ algorithm, which was not initially designed for large pairwise genome comparisons.

Locally performing large genome comparisons will often require long running times and most likely starvation of computational resources. Tools such as GECKO^10^, CGALN^11^ and MUMMER^12^ were created for large-scale comparisons of genomic sequences. However, computing a pairwise chromosome comparison usually takes from several minutes to hours depending on the length of the sequences, number of repetitions, and other factors, and the elapsed time increases quadratically when all chromosomes from two species are compared (i.e., *n* ∗ *m* comparisons). Furthermore, the previously mentioned algorithms spend most of their computation time processing at a certain degree of exhaustiveness, producing results such as complete alignments or collections of high-scoring segment pairs (HSPs). However, in the case of all *vs*. all comparisons, more than 400 chromosome comparisons (e.g. 20 * 20 chromosomes) can be involved. The resulting comparisons for a large deal of the dataset will often be composed of repeats and noise, with a low degree of conserved syntenies. Thus, it seems natural to incorporate a prior, heuristic step that analyses whether a particular comparison is of interest and reduces the computational complexity of performing the exhaustive comparison.

Our proposal focuses on a pre-processing, visualization and classification system to effectively determine whether conserved similarities exist across a collection of genomic sequences, therefore enabling the analysis without requiring long computation times. Within this framework, several post-processing tasks can be performed, such as detecting synteny blocks and large-scale genome rearrangements (LSGRs), tracing common ancestors of related organisms and placing new biological sequences on hierarchical trees.

In particular, we present a computational algorithm capable of extracting similarities from a pairwise genome comparison by indexing subsamples of inexact *k*-mers. Moreover, we also propose a heuristic and computational scoring metric to compute a scoring distance between sequences, which can be used to discard unrelated sequences in more exhaustive comparisons and hence speed up the comparison process. We show that our proposed method is able to perform large comparisons of plant genomes with up to 4.23 * 10^9^ base pairs in less than twenty minutes using one core and 1 GB of memory. In the case of mammalian organisms, our method can compare complete families of chromosomes from species such as *Homo sapiens* and *Mus musculus* (which amounts to 24 ∗ 21 = 504 comparisons) in less than 9 hours using only 1 core and 1 GB of memory. Our results are consistent and highly competitive (in terms of speed and quality) with other state-of-the-art software.

## Results and Discussion

**Figure 1:**
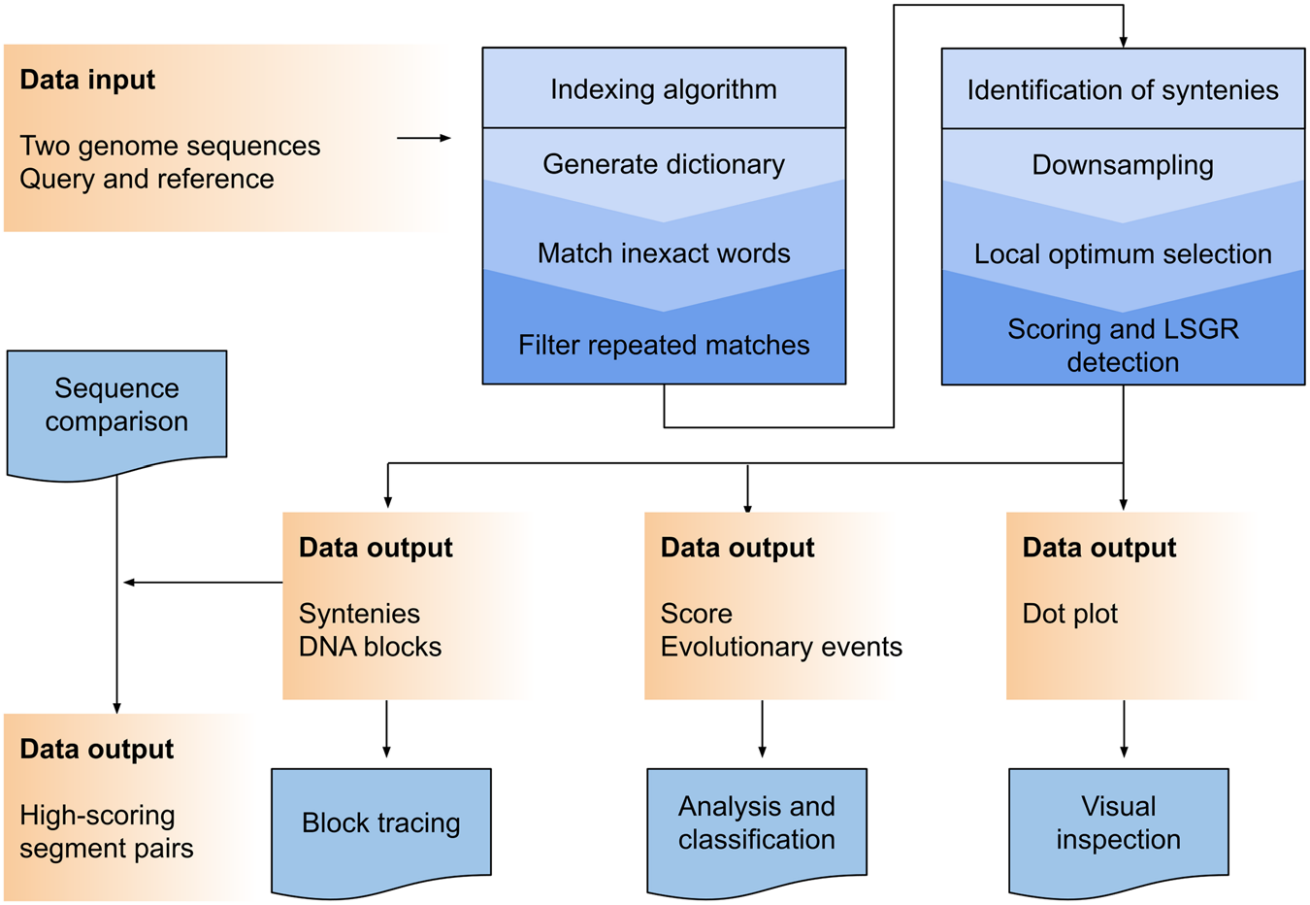
Workflow diagram of the proposed methodology. The query and reference genomes are compared in an ultra-fast, pairwise, coarse-grained fashion to ultimately produce a dot plot and a scoring distance and to detect large-scale genome rearrangements. A *k*-mer dictionary is built based on the query, and the reference *k*-mers are matched using an inexact subsampling procedure. Only unique matches are kept and recorded in a down-scaled representation (matrix) that groups matches to form larger fragments. Last, the dot plot is produced, a scoring distance is assigned to the comparison, and the LSGRs are reported. Additionally, using the DNA blocks produced by the method, users can employ further sequence comparison programs to generate exhaustive alignments.

This manuscript describes a linear computational algorithm for the ultra-fast comparison of large genomic sequences based on inexact *k*-mer matching. Briefly, the method proceeds by the following steps: (1) two DNA sequences are received as inputs; (2) a word dictionary is built based on the query sequence; (3) the words in the reference sequence are matched to those in the dictionary using inexact subsampling; (4) matched words are filtered based on uniqueness; (5) positional information is incorporated into a matrix representation, and the local optimum is found; and (6) a scoring distance is assigned to the comparison, and LSGRs are detected based on an iterative process. A workflow diagram of the process is depicted in Fig. 1.

Furthermore, a pipeline is provided for the exhaustive computation of conserved segments that re-uses the produced results. This method also outperforms current software in terms of the speed and is competitive in terms of quality of the results. See sections “Pipelined execution”, “Signal comparison” and “Sensitivity analysis and impact of the heuristic methodologies” in the Supplementary Material for a description of the methodology, quality assessment and further experiments. For information regarding the infrastructure employed in the comparisons, see “Infrastructure” in the Supplementary Material.

The proposed method is implemented in the C programming language under the name “CHROMEISTER”. It is able to compare very large genomes (due to its complexity being linear in time and constant in space in terms of the size of the sequences) much faster than state-of-the-art methods while yielding significant, reusable and exploitable information. The following experiments have been set up to show the capabilities of the method:

- Similarity search.
  - A very large pairwise comparison between the grass and common wheat genomes.
  - A synteny map of 12 primate species comprising full genomes.

- Evolutionary events across multiple species. Detected synteny blocks are employed to trace evolutionary events across species.
- Comparison of computation time and memory requirements with those of other methods.

### Very large pairwise comparison between the grass and common wheat (Aegilops tauschii and Triticum aestivum) genomes

**Figure 2:**
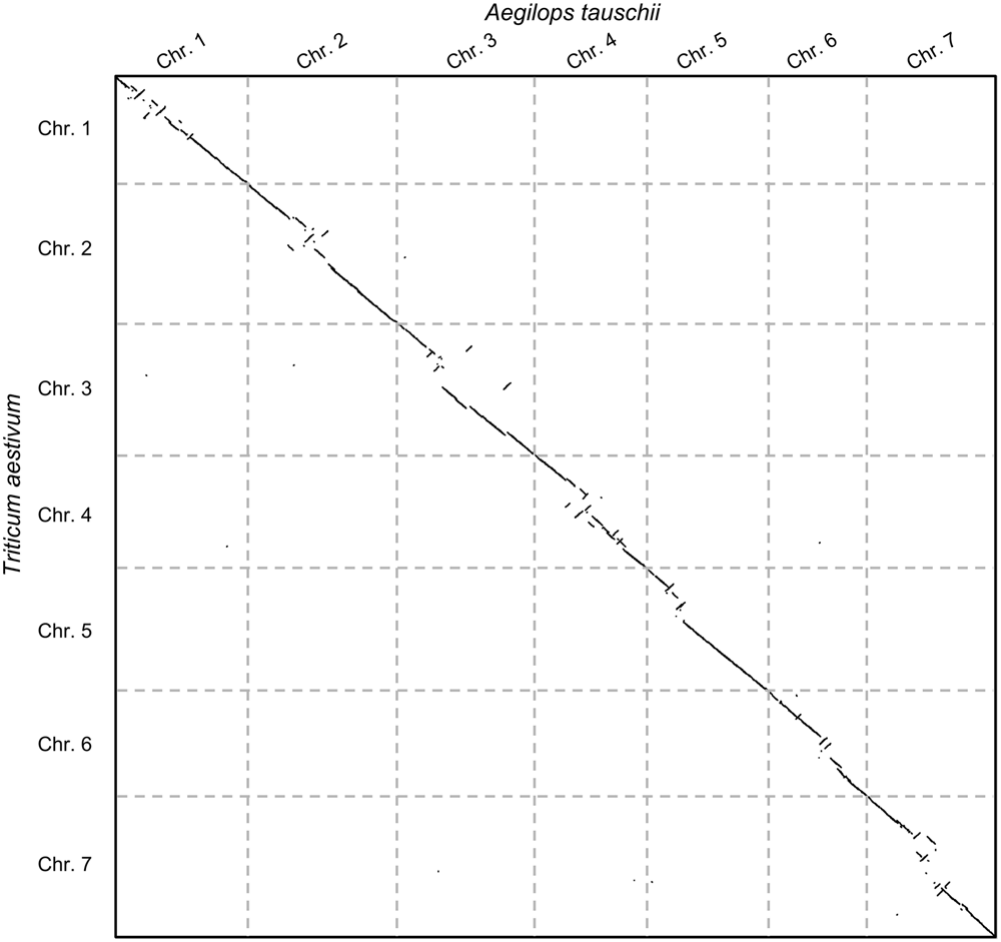
Pairwise genome comparison between *Aegilops tauschii* (3.2 Gbps) and *Triticum aestivum* (4.2 Gbps) performed in under 16 minutes using 1 core and 1 GB of RAM.

In general, plants are known to have sequences that are computationally difficult to compare due to their number of repeats^13^. We selected the fully assembled genome of two plants, namely, *Aegilops tauschii* (3.29 * 10^9^ base pairs) and *Triticum aestivum* (4.23 * 10^9^ base pairs), amounting to a search space of 1.39 * 10^19^ *bp*^2^ (see “Full genome sequences” in Supplementary Material). To the best of our knowledge, no such comparisons are available to serve as references. However, we reference several studies regarding the sensitivity of the proposed method in the Supplementary Material under the section “Sensitivity analysis and impact of the heuristic methodologies”. The genome sequences were obtained from the assemblies published in^14^ and^15^, respectively. Figure 2 shows the dot plot-like representation of the comparison. The computation took less than 16 minutes and 1 GB of RAM using 1 core, whereas the same comparison on NUCMER with parameters tuned to produce similar results took 159 minutes and 53 GB of RAM, which amounts to a speedup in terms of time consumption of over 10x while using 53 times less memory.

Only the main (or “unique”) signal can be seen in Fig. 2 due to the methodology automatically filtering out any DNA repeat or noisy signal. The comparison shows that nearly all conserved similarity is found on the diagonals and that each chromosome has only one pairwise comparison where a signal is found. This result accounts for an ideal scenario in which the application of the method can reduce the complexity of an exhaustive comparison since each chromosome would need to be compared only once (yielding *n* comparisons) as opposed to an all vs all approach, which requires a quadratic number of comparisons (i.e., *n* * *m*), hence dropping the complexity from quadratic to linear in terms of the number of comparisons. Notably, although it seems that no synteny occurs for any of the other chromosomes, a very large number of repetitions are present that would still require computing power to process.

In the further scenario of an all vs. all comparison among several species (e.g., all chromosomes of all genomes from a taxonomic family), this reduction enables a significantly faster approach to obtaining the conserved syntenies. Moreover, the process is completely automatic since the scoring function determines which comparisons have a signal and therefore removes the tedious task of manually inspecting up to thousands of comparisons by hand (e.g., there are 6,000 comparisons for 5 species with 20 chromosomes each since for *s* species and *n* chromosomes, we have $\frac{1}{2}s(s+1)\ast n\ast n$ comparisons).

Figure 2 was constructed from a full-genome comparison (as opposed to a chromosome-by-chromosome comparison), which yielded a 0.28 score (with 0 indicating the exact same sequences and 1 indicating absolutely no similarity, see “Methods” for more on the scoring distance). Such a score is due to both genomes conserving most of their diagonal, but small gaps still exist along with several evolutionary events that result in scoring penalties.

### Synteny map

**Figure 3:**
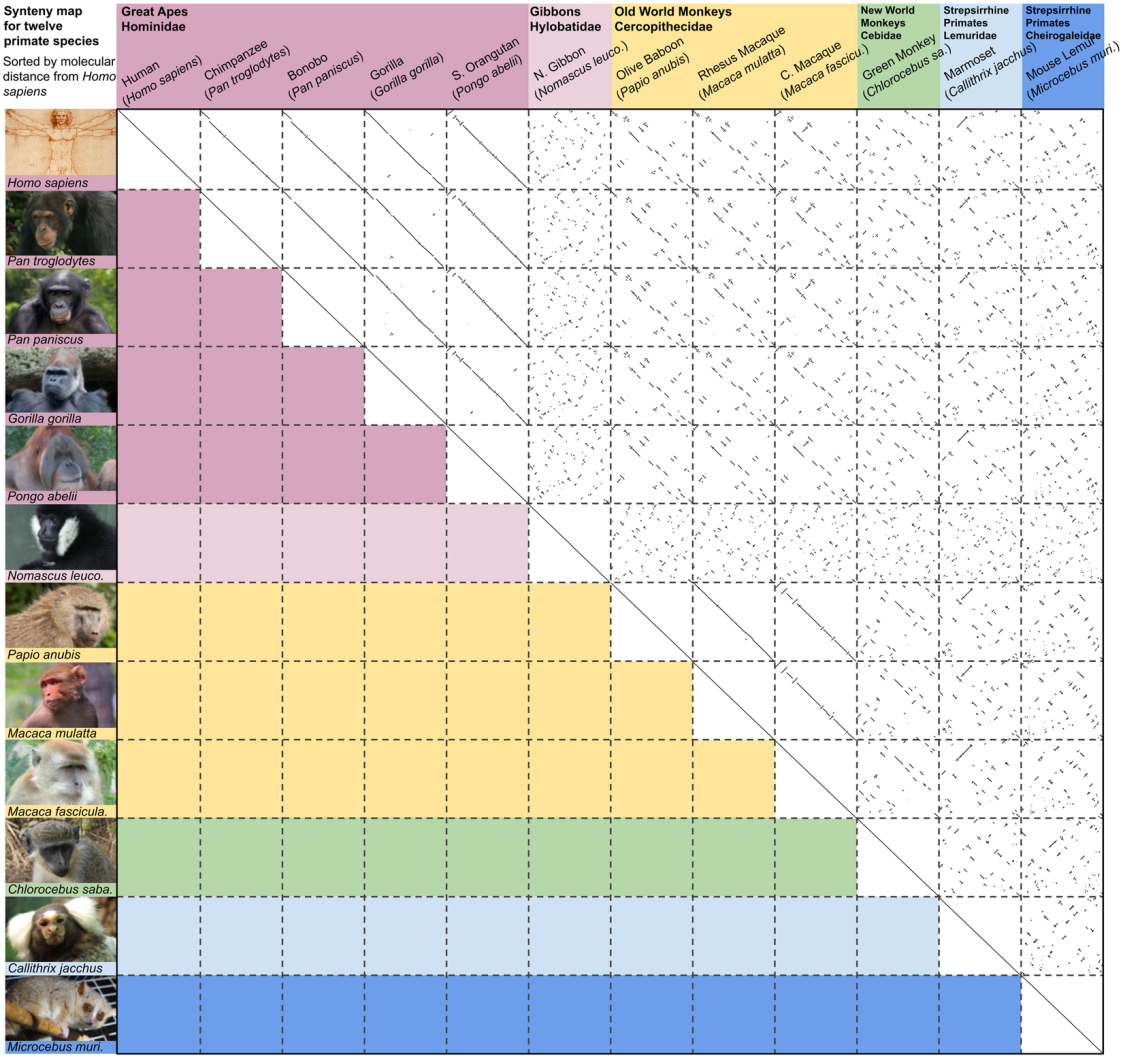
Similarity map of twelve primate species. Full genomes were compared. From left to right and top to bottom, genomes are ordered using the maximum likelihood method based on amplified genes. The compared genomes belong to six families, i.e., Hominidae (dark purple), Hylobatidae (light purple), Cercopithecidae (yellow), Cebidae (green), Lemuridae (cyan) and Cheirogaleidae (blue). Each cell of the upper right matrix shows the dot plot corresponding to the comparison of the row and column. The list containing copyright permissions of the pictures used in the Figure is available in the Additional File 1.

The proposed method is suitable for performing very large all versus all comparisons, from bacterial collections to full genomes. In this section, we illustrate a comparison featuring 12 primate full genomes. The sequences were obtained from the Ensembl database^16^. The collection includes the fully assembled genomes of *Homo sapiens*, *Pan troglodytes*, *Pan paniscus*, *Gorilla gorilla*, *Pongo abelii*, *Nomascus leucogenys*, *Papio anubis*, *Macaca mulatta*, *Macaca fascicularis*, *Chlorocebus sabaeus*, *Callithrix jacchus* and *Microcebus murinus*. These species were selected because (1) they belong to different evolutionary branches of primates and (2) they were fully assembled. The order in which these sequences are presented is based on their evolutionary distance from the reference (*H*. *sapiens*), as depicted in^17^, which was determined by the maximum likelihood method based on 34,927 base pairs sequenced from 54 amplified genes. The computation of the $\frac{1}{2}n(n+1)$ full genome comparisons took 23 hours and 21 minutes using 1 core and 1 GB of RAM, meaning that each comparison took, on average, less than 18 minutes. The synteny map is shown in Fig. 3.

Figure 3 shows how the proposed method can be used to generate very large synteny maps that enable inspection of conserved similarities and evolutionary events among multiple species. Furthermore, analysis of the similarity map leads to several points of discussion: (1) similarity is mostly conserved across all species, although the number of evolutionary events increases as a function of the molecular distance; (2) closely related families such as the great apes (Hominidae) and the Old World monkeys (Cercopithecidae) show nearly no large-scale genome rearrangements except for a few inversions; and (3) although according to several techniques (such as maximum likelihood-based^18^ and mitochondrial DNA-based^19^ techniques), *N*. *leucogenys* is placed on the branch closest to Hominidae (namely, Hylobatidae), the opposite case occurs from an LSGR perspective. In fact, all of the Old World and New World monkeys, along with the marmoset, have fewer LSGRs than *N*. *leucogenys* with respect to *H*. *sapiens* (See “Detected LSGRs in the synteny map“ in the Supplementary Material). That is, except for the case of *N*. *leucogenys*, the comparisons exhibit a correlation between molecular distance and the number of LSGRs.

However, it is beyond the scope of this manuscript to answer these questions; instead, we offer and describe a new tool capable of producing this type of highly aggregated and coarse-grained information in a few hours using one core, thus providing the means to obtain new findings and perform novel research.

### Evolutionary events across multiple species

In this section, we illustrate how the developed method can be employed in evolutionary studies. In particular, a simple synteny block-tracing algorithm is devised based on the location of large collections of HSPs. That is, given an all *vs*. all comparison among several species, with CHROMEISTER, it is possible to trace conserved blocks across species. In this example, we choose a conserved block from a particular species and trace it, and whenever an overlapping conserved region is found, another tracing procedure is launched from that particular block to examine the remaining species. The order in which executions are launched does not affect the outcome, as species can be interchanged freely. Therefore, a tree-like representation is generated that is rooted in the initial block and origin species, with every internal node being an overlapping match with another species. In this particular case, we employ *H*. *sapiens* chr. 6 as the root of the tracing algorithm and trace it to a 5-species depth.

**Figure 4:**
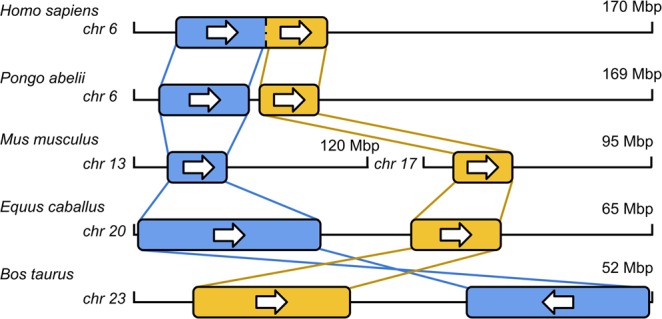
Tree-tracing representation of the evolutionary study. Large groups of HSPs are shown as synteny blocks on each chromosome. Traversing lines from one chromosome to another represent synteny in terms of conserved HSPs. Crossed lines imply that a reversion occurred.

As shown in Fig. 4, an initial block of conserved HSPs from *H*. *sapiens* chr. 6 first split into two blocks in *P*. *abelii* chr. 6, which then split into two different chromosomes from *M*. *musculus*, namely, chromosomes 13 and 17. These two blocks reunite in chromosome 20 from *Equus caballus* and are finally detected on chromosome 23 from *Bos taurus*. The order (rooted from *H*. *sapiens*) has been determined according to the vertebrate mitochondrial phylogeny described in^19^. Note that in Fig. 4, only the blocks fully traced from the root (*H*. *sapiens*) to the leaf (*B*. *taurus*) are shown, i.e., there is a larger synteny block between chromosome 6 of *H*. *sapiens* and *P*. *abelii*, but it does not reach the leaf.

For further details on how the tracing of blocks was performed, see “Block tracing” in the Supplementary Material.

### Comparison of computation time and memory requirements with those of other methods

In this section, we compare the performance of the proposed method with that of other state-of-the-art software in terms of time consumption and memory requirements. To make the comparison as fair as possible and to yield the same levels of sensitivity, both CGALN and NUCMER were tuned to run faster and produce coarse-grained results (see “Software Parameters” in the Supplementary Material). The sequences employed were selected based on increasing size and ranged from single chromosomes (e.g., B19-C9 stands for *B*. *taurus* chr. 19 and *Canis familiaris* chr. 9) to complete genomes composed by all chromosomes (for example, see HC-MC). Further details on the compared sequences can be found in the Supplementary Material (see “Full genome sequences” and “Additional Table 1”).

Several observations can be made from Table 1. Note that in the case of CHROMEISTER, the runtime is linearly correlated with the search space, whereas in the case of CGALN or NUCMER, other factors must be considered (for instance, BC-BC takes longer than A1-T1), such as the number of repetitions and total number of seeds and hits matched. In terms of performance, CHROMEISTER is the fastest (up to 11 times faster than NUCMER for the largest dataset), using linear time in terms of the size of the sequence input and a constant memory footprint. In contrast, CGALN results in incomplete executions after 100 hours of computing time for the last three datasets. NUCMER is able to finish all executions in competitive time but requires a much larger amount of RAM (up to 50 times more) than CHROMEISTER. Moreover, such memory usage poses computational limits, both for regular, desktop computers, and even in the case of supercomputers when several executions are to be run in parallel.

**Table 1:** Comparison of the best performances of CGALN, NUCMER and CHROMEISTER.

Dataset	Search Sp. (*bp*^2^)	CGALN		NUCMER		CHROMEISTER	
		Time (m)	RAM (MB)	Time (m)	RAM (MB)	Time (m)	RAM (MB)
B19-C9	$4.03\ast {10}^{15}$	1.63	755	2.13	812	**0.89**	**994**
HX-MX	$2.07\ast {10}^{16}$	5.93	2,032	5.14	2,221	**1.29**	**998**
BC-BC	$1.18\ast {10}^{17}$	141.26	583	13.61	5,814	**2.42**	**1,022**
M2-O1	$1.54\ast {10}^{17}$	17.04	3,618	15.14	6,436	**2.84**	**1,017**
A1-T1	$2.57\ast {10}^{17}$	DNF	DNF	11.40	8,357	**2.84**	**1,022**
HC-MC	$8.69\ast {10}^{18}$	DNF	DNF	168.86	46,091	**13.77**	**1,022**
AC-TC	$1.39\ast {10}^{19}$	DNF	DNF	176.80	52,021	**15.96**	**1,022**

The datasets are sorted by search space size. Columns from left to right: the sequences compared, search space size, time (in minutes) and memory consumption (megabytes) for each method. The acronym “DNF” stands for “did not finish”, which represents more than 100 hours of computing time. The time reported by NUCMER indicates that the program used more than one CPU; therefore, the adjusted time is shown.

Finally, it is important to note that CHROMEISTER detects the main DNA segments and therefore does not report repetitions, as in the case of NUCMER or CGALN. This difference has a clear impact on performance due to algorithmic improvements that can be made and in terms of sensitivity since repeated regions are discarded. For a sensitivity comparison, please see the section “Sensitivity analysis” in the Supplementary Material. In addition, a comparison of fine-grained alignments between the methods and the CHROMEISTER pipeline is also available in the section “Pipelined execution”.

## Conclusions

In this manuscript, we have developed a heuristic method for quickly determining conserved similarities in very large genome comparisons using unique and inexact hits. We have also shown that the time complexity of the method is linear in terms of the size of the input sequences, whereas the space complexity is constant, making the method suitable for virtually any comparison of nucleotide sequences. In particular, the method described is intended to extract conserved diagonals across species by applying probabilistic filters that remove traces of noise and repeated DNA segments. However, the purpose of the algorithm is not limited to its primary use (detection of conserved diagonals) but extended to other analyses and studies, such as the following:

1. In terms of data exploration, the method can be used to quickly obtain a previsualization dot plot of a large set of sequences, which helps the researcher obtain a quick overview and even perform basic quality control steps.
2. Regarding multiple sequence alignment (MSA), the method can be employed to generate the anchoring distance matrix to guide hierarchical MSA by using the coverage-based scoring system.
3. In terms of search space, the method is suitable for strongly pruning the search space that arises from the sequence comparison since noise and DNA repeats are removed. This pruning enables the algorithm to keep only information (which can be used in later post-processing stages) about the synteny blocks. For instance, the algorithm is well suited as a prior step to a whole-genome alignment experiment.
4. For evolutionary studies, the proposed method is able to detect LSGRs throughout collections of species, which can be used to trace evolutionary events, as shown in the example cases.
5. For phylogenetic studies, the method can be employed to incorporate LSGRs into combined phylogenies.

The extension of capabilities of the proposed method is performed by pipelining an exhaustive comparison that takes advantage of the information generated in the fast comparison and hence reduces running times and enables further post-processing, such as detection of syntenies and evolutionary events. Furthermore, the pipelining method enables extremely large comparisons on the order of several gigabases to be performed quickly, using regular desktop computers and with a high degree of exhaustiveness.

From the computational perspective, the proposed method is linear in time and constant in space with respect to the input sequences. These features imply that the runtime increases linearly with the size of the sequences while the memory requirements remain constant. Furthermore, its parallelisation becomes immediate through a map-reduction strategy (see Supplementary Material, section “Parallel execution”).

Additionally, we have proposed a simple, coverage-based scoring distance that is able to evaluate a comparison based on the cleaned conserved signal, which can be used for evaluation purposes, particularly to discard comparisons where no similarity can be found and hence avoid expensive and exhaustive computational processing. This metric can also be aggregated to generate other indicators of the quality of comparisons.

## Methods

In the following sections, we introduce the working methodology of the proposed algorithm. In particular, the following subsections are considered:

- Previous definitions. Several definitions will be made to handle descriptions with a higher degree of rigor.
- Inexact *k*-mer indexing. The process of indexing inexact *k*-mers is described regarding the hashing function used to index them.
- Heuristic filtering. The filtering method used to separate repeats and noise from conserved similarities is described in this subsection.
- Scoring function. The scoring function employed to classify genomic sequences depending on their closeness is explained.
- Implementation. Computational details regarding the deployment of the algorithm are described.

### Previous definitions

In this section, we introduce definitions, expressions and mathematical distributions to model a scenario for DNA comparison. In particular, DNA words, the matching of these matches from one sequence to another and the distributions that govern these matches are defined.

Consider a finite alphabet $S=\{A,C,G,T\}$. A word *w* is defined as any finite string of letters in the alphabet *S*, i.e., *w* is generated by the regular expression [*ACGT*]+. The set of all words *w* over *S* will be denoted *U* (the universe). We will define a sequence *s* as a word of size *n*, that is, $|s|=n$. The collection of words *w* of size *k* contained in *s* is denoted $C=\{{w}_{1},{w}_{2},\ldots ,{w}_{m}\}$, where *m* = *n* − *k* + 1, which accounts for all possible overlapping words of fixed size *k* in the sequence *s*.

We will define the collection of similar segments between two sequences *s*_*a*_ and *s*_*b*_ as ${H}_{a,b}\subseteq {C}_{a}\times {C}_{b}$, which accounts for the words shared (*w*_*i*_, *w*_*j*_) by *C*_*a*_ and *C*_*b*_ (also known as “hits”). The distribution of the set *H*_*a*,*b*_ is highly dependent on the two sequences *s*_*a*_ and *s*_*b*_ that produce it since more similar sequences will produce more similarities, whereas distant sequences will produce fewer similarities. The *k* size of the words will also strongly affect the set *H*_*a*,*b*_ (for a detailed analysis of the word distributions, see^20^).

Moreover, we will further re-define, from a coarse-grained perspective, *H*_*a*,*b*_ to be the composition of three other distributions, namely:

- *W*_*a*,*b*_ is the distribution of the random hits between *s*_*a*_ and *s*_*b*_. Given a sufficiently large *n*_*a*_ and *n*_*b*_ (lengths of the two sequences, respectively), the probability of finding random hits follows a binomial distribution (assuming uniformity of the letters in the sequences) $B(|{C}_{a}\times {C}_{b}|,{\frac{1}{4}}^{k})$. For further clarification of this distribution, see the section “Distribution of hits” in the Supplementary Material. Hence, *W*_*a*,*b*_ is the distribution of pure random noise (therefore originating by chance).
- *R*_*a*,*b*_ is the distribution of any hit such that its words *w*_*i*_, *w*_*j*_ are scattered repeatedly (i.e., frequency greater than one) across both sequences *s*_*a*_ and *s*_*b*_ and/or their complexity (measured as the longest unique substring contained in a word *w*, see^21^) is considered to be low based on biological standards. Furthermore, from a coarse-grained perspective, this distribution accounts for any repeatable element, such as tandem repeats, low-complexity regions, and interspersed repeats.
- *T*_*a*,*b*_ is the distribution of the signals conserved between *s*_*a*_ and *s*_*b*_ such that ${T}_{a,b}={H}_{a,b}\backslash ({W}_{a,b}\cup {R}_{a,b})$. That is, hits whose words *w*_*i*_,*w*_*j*_ are unique (i.e., their frequency is one) are shared with respect to a common ancestor (conserved), did not originate by chance and cannot be considered repeats under biological examination.

### Inexact k-mer indexing

In this section, we describe the procedure by which the words are matched in an inexact fashion using heuristic subsampling of all possible matching combinations.

A perfect indexing hash function *f* for the universe *U*^*k*^ (i.e., all existing words *w* of size *k* over *S*) is given by the function:

1 $$\begin{array}{rcl}f({w}_{i}^{k}) & = & \,{|S|}^{0}\ast v({w}_{i,1}^{k})+{|S|}^{1}\ast v({w}_{i,2}^{k})+\ldots +{|S|}^{j-1}\ast v({w}_{i,j}^{k})+\ldots +{|S|}^{k-1}\ast v({w}_{i,k}^{k})\\ f({w}_{i}^{k}) & = & \sum _{j=1}^{k}{|S|}^{j-1}\ast v({w}_{i,j}^{k})\end{array}$$

where ${w}_{i,j}^{k}$ denotes the *j*-th letter of a word *w*_*i*_ with $|{w}_{i}|=k$ and function *v*(*x*) returns the integer position of *x* in *S*, starting from zero (i.e., *v*(*A*) = 0 and *v*(*T*) = 3). Note that function *f* is a bijection $f:{U}^{k}\to {N}^{\{1..{|S|}^{k}\}}$ that creates perfect indexing with no collisions for *U*^*k*^; thus, the number of images in the codomain is equal to that in the domain, which accounts for 4^*k*^. Furthermore, we will define the family of functions *F* as the collection of functions $F={\cup }_{z=1}^{\infty }f{(x)}^{z}$ where $$\begin{array}{lll}f{({w}_{i,j}^{k})}^{z} & \,= & \sum _{j=1}^{k}({|S|}^{j-1}\ast v({w}_{i,j}^{k})\ast belongs(j,z))\end{array}$$, and the operation $belongs(j,z)$ returns 1 if there exists a natural number *x* such that $j=z\ast x$ (that is, *j* is a multiple of *z*) and otherwise 0. Therefore, the collection of functions *F* contains all indexing functions *f*(*x*)^*z*^ that use either all letters, half of the letters, or a third of the letters and so on (intermittently) to compute the hash of *w*. That is, the higher the *z* value of the *f*(*x*)^*z*^ function is, the larger the number of collisions will be. Ideally, every skipped letter would increase the number of collisions by $|S|=4$ since ${w}_{i,j}^{k}$ can take up to 4 different values at position *j*. Using any of the functions *f*(*x*)^*z*^ with *z* > 1 will produce inexact indexing, enabling the detection of long, imperfect matches that also incorporate positional information within a *k*-mer. As opposed to using smaller *k*-mers, inexact *k*-mers are not as prone to the generation of random noise in a comparison. Therefore, any indexing function taken from the family *F* (with *z* > 1) will enable heuristic subsampling of all possible inexact *k*-mer matches. Determining whether *k*-mers are similar but inexact at one position requires 3^*k*^ comprobations in a brute force approach. The complexity of doing so grows exponentially with the length of *k* and the number of inexactitudes allowed. Thus, by using the *F* family of functions, the search for inexact *k*-mers is performed in *O*(1) time heuristically (see Supplementary Material for more details).

### Heuristic filtering

In this section, we describe the methodology by which repetitions and noise are filtered out of the main signal.

Given a sufficiently large *k* and an indexing function *f*, the hits produced by the distribution *W*_*a*,*b*_ (i.e., random hits) can be ignored, as the probability of finding random matches between sets of words *C* and *C*′ of length *k* and set sizes *m* and m′ (respectively) is $1-{({(1-\frac{1}{{4}^{k}})}^{m})}^{m^{\prime} }$ (see Supplementary Material, section “Random perfect hits”), amounting to *p* = 0.0005419 for the case of mammalian chromosomes of up to 100 million base pairs and *k* = 32.

To remove the noise generated by *R*_*a*,*b*_, only the unique hits in *H*_*a*,*b*_ are kept. By definition, a repetition is any word *w*_*i*_ whose frequency is greater than one. Therefore, removing all hits (*w*_*i*_, *w*_*j*_) from *H*_*a*,*b*_ where *w*_*i*_ appears more than once will effectively remove all possible repeats belonging to the distribution *R*_*a*,*b*_. However, exact words tend to be rarer as *k* becomes larger (due to millions of years of evolution, see^22^), and a small *k* can produce random noise. To overcome this scenario, a hybrid approach is employed: a large *k* of 32 with a heuristic indexing function (*z* > 1, as explained before), which enables finding long, unique words with still some degree of variability. The impact of *z* on the number of unique inexact hits found (and therefore employed to detect similarity) is analysed in the Supplementary Material.

Nonetheless, this approach is not sufficient to determine the conserved similarities since even distantly related organisms *a* and *b* will have a substantial number of small conserved signals (e.g., basic functioning genes, see^23^). To extract the conserved similarities, it is necessary to group (cluster) the hits that belong to long, shared portions of the genome (i.e., HSPs) instead of just conserved genes. To do so, downsampling is applied, i.e., the position of hits is heavily scaled down to force hits belonging to an HSP to fall in the same position, hence increasing the concentration of hits in regions where syntenies are to be found. This approach is similar to two-dimensional positional clustering of the hashes identified during the matching steps (see, for example, MOSAIK^24^, which employs positional clustering of hashes to consider candidate regions to be aligned).

**Figure 5:**
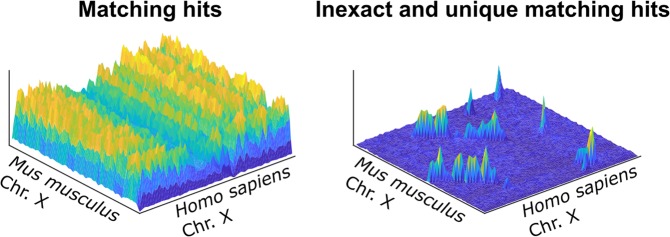
Three-dimensional histograms of the hits matched using either an exact procedure (left) or the heuristic method proposed (right). The comparison was performed between the X chromosome of the human and mouse genomes.

Formally, consider an HSP to be a subset of the collection of words *C*_*a*_ and *C*_*b*_, that is, for some starting positions *i*, *j* and for a given length, the words *w*_*i*_ and *w*_*j*_ in *C*_*a*_ and *C*_*b*_, respectively, are equal (notice that not all words have to be equal, i.e., mismatches can occur). Then, it is clear that linear downsampling will map hits near position *i* together into a new position *i*′ if a uniform surjective mapping function is used since clusters will form from nearby hits that belong to conserved signals. The very same outcome is observed for *j*. Therefore, a larger number of hits will be mapped to positions *i*′ and *j*′ if an HSP exists, which enables it to be detected as a conserved signal. Figure 5 shows an example comparison of an exact word-matching procedure and the heuristic method described. Note that in the exact procedure, the conserved HSPs are outnumbered by repeats and thus remain undetected. However, in the heuristic procedure, only the conserved HSPs are kept, while repeats are removed.

### Scoring function

In this section, the function used to score a pairwise comparison between two sequences is described. Such a function is presented as a heuristic discriminant of related from unrelated comparisons.

Once the surjective function is applied and the comparison is mapped to a lower-scale dimension, the highly conserved diagonals are sought. We will call the new dimension the hit matrix *H*_*m*_, which is built by counting the found inexact hits and their scaled positions and represents a footprint of the comparison. Since conserved signals are diagonal in nature (i.e., hits belonging to an HSP are adjacent in terms of the diagonal in a 2D cartesian plane, such as in a dot plot representation), they are extracted by finding the maximum peak of concentrated hits per row in *H*_*m*_. That is, there is no overlapping of conserved signals in *H*_*m*_, as doing so would include repetitions. The scoring function then sums the distances between the conserved signals using the taxicab distance and scales it between [0, 1], with 0 indicating a perfect diagonal (i.e., identical sequences are compared) and 1 indicating completely different sequences with no conserved regions. Formally, the scoring function is calculated as follows:

2 $$\begin{array}{c}{d}_{raw}=\sum _{i}^{l-1}taxicab({\rm{\max }}({H}_{m}(i),{H}_{m}(i+1)))\,\end{array}$$

where *l* is the longest dimension of *H*_*m*_. Note that this distance takes into account large genome rearrangements such as transpositions or inversions since every gap between conserved signals is penalized. However, *d*_*raw*_ accounts only for the sum of the taxicab distance between the continuous conserved signals and needs to be normalized. This normalization is carried out by dividing *d*_*raw*_ by the theoretical maximum distance, which accounts for a scenario in which conserved signals are intermittently found on opposite sides of the sequences, namely:

3 $$d=\frac{{d}_{raw}}{{l}^{2}}$$

where *d* accounts for the normalized distance (or score) between sequences *a* and *b*. Further details on the aggregation of the metric for split genomes (such as those divided between chromosomes) can be found in the Supplementary Material under the section “Distance aggregation”.

### Implementation

The algorithm described above is intended to be used heuristically in fast, hit-based sequence comparison approaches. In short, the full algorithm comprises the following:

1. Indexing the unique inexact *k*-mers of the reference sequence.
2. Computing the hits between the reference and query sequences.
3. Removing non-unique hits.
4. Downsampling the hits matrix *H*_*m*_.
5. Filtering by finding the maximum peaks.
6. Computing the distance between the sequences.
7. Detecting LSGRs.

The most demanding computational step is the indexing of *k*-mers (retrieval is performed in constant time since only unique hits are kept). To accelerate this process, indexing involves two different mechanisms. Words are hashed and inserted into a hash table of size 4^12^ (determined empirically as a good trade-off between memory consumption and sensitivity), where each entry corresponds to the 12-nucleotide prefix of each word (similar seed patterns have already been explored^25^). A posterior search is performed by first accessing the hash table entry corresponding to the word prefix (*O*(1)) and then comparing the hash key of the stored unique *k*-mer with the target key. Downsampling is performed by linearly mapping each hit *h*_*i*_ to a new position *i*′ in the hit matrix *H*_*m*_. Note that the larger the *H*_*m*_ matrix becomes, the higher the resolution for mapping hits becomes; however, this increase occurs at the expense of space consumption, which in this case accounts for second storage memory. The resolution of the matrix can be chosen based on user interest since a higher resolution will allow more detail, whereas a lower resolution will show a more coarse-grained view of the comparison.

Since the output of the algorithm is a dot plot representation of the comparison (intrinsically a matrix of integers), the detection of LSGRs is analogous to detecting lines in an image. Extensive literature is available on this subject (see, e.g.)^26,27^, which is beyond the scope of this manuscript. In this particular case, we employed a parameterized growing-region algorithm that is launched for every local optimum in the matrix and for which the edge direction is weighted depending on the angle of nearby hits (i.e., diagonals and anti-diagonals are prioritized over perpendiculars). For further details, see the Supplementary Material.

### Equipment and settings

All figures (except the diagram in Fig. 1) were built from data generated from the proposed method CHROMEISTER. Figures 2, 3 and 4 were put together with Google Drawings and Inkscape. Figure 5 was generated using MATLAB. GIMP was employed in Figs 2 and 3 to improve edges visualization, particularly in the small sized thumbnails in Fig. 3.

### Third party rights

Figure 3 includes several photographs of Apes, Gibbons, Monkeys, etc. Copyright is available for all of these pictures. The only changes made to these images were partially cropping. The list containing copyright permissions of the pictures used is available in the Additional File 1.

## Supplementary information

Supplementary Material

Additional File 1

## Data Availability

All data generated or analysed during this study are included in this published article (and its supplementary information files).

## Code Availability

All sources, scripts and executables described or employed in the manuscript can be accessed at https://github.com/estebanpw/chromeister.
