# Supplementary material for: Ultra-fast genome comparison for large-scale genomic experiments
Source: Sci Rep. 2019 Jul 16;9:10274. doi: 10.1038/s41598-019-46773-w (PMC6635410; doi:10.1038/s41598-019-46773-w)
Supplement: Supplementary file 1 — Supplementary Material [file 41598_2019_46773_MOESM1_ESM.pdf]

Ultra-fast genome comparison for large-scale genomic experiments

Supplementary Material

Esteban Pérez-Wohlfeil[1], Sergio Diaz-del-Pino[1] and Oswaldo Trelles[1]

1. Computer Architecture Department, University of Málaga - Instituto de Investigación Biomédica de Málaga-IBIMA, Spain

Glossary **2**

Novelty of the method **3**

Full genome sequences **3**

Sensitivity analysis and impact of the heuristic methodologies **3**

Gene search in a closely related scenario 3

Gene search comparison with other methods 5

Indexation and matching procedure 6

False positives and false negatives due to inexactness 7

False positives and false negatives due to uniqueness 8

Regarding error susceptibility of inexact versus traditional k-mers 9

Pipelined execution **11**

Signal comparison **12**

Infrastructure **14**

Detected Large-Scale Genome Rearrangements in the synteny map **14**

Correlation between LSGRs and the scoring distance **15**

Block tracing **17**

Parallel execution **18**

Distribution of hits **18**

Impact of inexact indexing **19**

Plant genomes and uniqueness **20**

Further visualization **21**

Comparison M2-O1 22

Comparison BC-BC 23

Comparison HC-MC 24

Comparison AC-TC 25

Software parameters **25**

NUCMER CPU usage **26**

Single One vs. One chromosome comparison and software comparison **26**

Proof for new fragments detected between Bos taurus 19 and Canis familiaris 9 **29**

Proof for new fragments detected between Homo sapiens X and Mus musculus X. **31**

NUCMER comparison **35**

Pipelined execution **36**

Random perfect hits **43**

Additional Table 1 **44**

Additional Table 2 **45**

Additional Table 3 **46**

Coverage comparison using MEGABLAST **46**

Distance aggregation **46**

Poisson distribution in split number of chromosomes **47**

List of copyright permissions for the pictures shown in Figure 3 **50**

References **51**

Glossary

Dotplot: A comparison between two sequenced represented in the Cartesian plane.

Hit: An identified match between two *k*-mers.

Indexing: In the context of *k*-mers, computing a hash (footprint) of the *k*-mer to store it in order to improve posterior retrieval.

Inexact k-mer: A k-mer that has been indexed using a key which represents the *k*-mer partially to force collisions.

K-mer: A DNA substring of fixed size *k*.

Repeats: A DNA string that appears repeatedly in a DNA sequence, typically ranging from few base pairs to a few thousands.

Search space: Set containing all possible solutions for a given problem.

Subsampling: In the context of *k*-mers, selecting a subset of all *k*-mers according to an heuristic.

Synteny: Two or more blocks present in species being compared which conserve relative order.

Novelty of the method

The study of different seed patterns for the matching of *k*-mers has been studied several times. For instance, in "Bin Ma, John Tromp and Ming Li. 'PatternHunter: faster and more sensitive homology search.' Bioinformatics 18.3 (2002): 440-445" different patterns and their optimality for sensitivity are explored. In our approach we employ a different seed pattern that can be changed as a function of the Z value in order to be able to change it as required depending on the relatedness of the sequences being compared. Therefore, strictly speaking, it is not a novel method on its own. However, the inexact approach is not the only methodology in the algorithm - moreover, the proposed algorithm is a combination of several heuristics:

- Inexact matching to cope with DNA mutation, sequencing errors, etc.
- Uniqueness to reduce the search space while removing repetitions.
- Subsampling and compression to amplify the signal to noise ratio.
- Local maxima traversing of high concentration points (peaks) of hits to select conserved diagonals.

The combination of these is what brings novelty into the approach, ultimately resulting in a very fast algorithm to pre-visualize huge comparisons, filter noise and repeats, detect synteny blocks, etc.

Full genome sequences

In some experiments (e.g. Figure 2 or 3 in the manuscript) the complete genomes were built from their chromosomes. This procedure involved creating a large FASTA file with all chromosomes concatenated one after another. The order was decided by length (longer chromosomes first). This scenario enables basically to generate a large synteny map where all chromosomes are compared at once, and carries no extra biological significance. Moreover, it is also much faster, although some information is lost since more is compressed into less space.

Sensitivity analysis and impact of the heuristic methodologies

Gene search in a closely related scenario

Indexing only unique and inexact *k*-mers will carry a cost in sensitivity. However, in the case of homologous genes which share a large deal of their *k*-mers, as long as there are some *k*-mers not shared between genes (even with one) it will contribute to detecting homology in the particular region. This is due to the number of matched *k*-mers being grouped together to form larger fragments. Regions that do not have any homology will very rarely include a matched *k*-mer (and therefore no fragment will be formed), but regions sharing homologous genes have the potential to include also unique *k*-mers that will be matched afterwards. Probabilistically speaking, and since the method is aimed for large comparisons:

1. Regions with no homology will form no fragments as there are nearly no *k*-mers to be matched.

2. Regions with homologous genes will form fragments as these include more *k*-mers to be matched than the previous case (even though not many will be unique).
3. Regions with repeats will form no fragments as nearly all of their matching *k*-mers will be removed due to uniqueness.

We have performed an experiment to measure the sensitivity of the method regarding genes found in more closely or distantly related genomes, particularly for the Escherichia Coli, whose relation among strains is closely depicted in [1]. Therefore we downloaded the strains B, K-12, O157:H7, *Shigella flexneri2a* and CFT073 (in order of relatedness regarding the B strain). These were all compared to the B strain using first CHROMEISTER and then the pipeline (i.e. GECKO guided by the information generated with CHROMEISTER). Then, all genes found in the comparison for each sequence were counted for the forward strand. The sensitivity was calculated as follows:

$$S = \frac{TP}{TP+FN} = \frac{genes\ identified}{total\ number\ of\ genes\ in\ the\ comparison}$$

Notice that in order to calculate the sensitivity we need a ground truth reference, which in this case, will be GECKO. Therefore, the sum of the true positive and false negative rate, which equals the total number of genes in the comparison will be provided by GECKO, and the reported results by the CHROMEISTER pipeline will be calculated upon these. Table 1 shows the results in sensitivity for the depicted comparisons.

Comparison	Query cover MEGABLAST	Genes found in the B strain	Genes found in the alternative strain
B vs. K-12	94%	1912 / 2096 (91%)	1915 / 2077 (92%)
B vs. O157:H7	91%	1720 / 2002 (85%)	1581 / 1861 (84%)
B vs. *Shigella flexneri* 2a	84%	1329 / 1613 (82%)	1222 / 1568 (77%)
B vs. CFT073	88%	1577 / 1940 (81%)	1511 / 1847 (81%)

Table 1. Genes detected (sensitivity) in the comparisons of the strain Escherichia coli B with different strains. The divisor in each cell represents the total number of genes in the comparison as provided by an exhaustive GECKO search. The dividend corresponds to the true positive rate as identified by the proposed method.

We can observe from Table 1 that the results are consistent with those reported in [1] and that moreover, the CHROMEISTER pipeline only loses a relatively small percentage of the signal when the relatedness between sequences lowers.

Gene search comparison with other methods

We have performed a similar experiment to the previous one to measure how many genes are detected comparing other software, particularly NUCMER, GECKO and the CHROMEISTER pipeline (that is, using the information generated by CHROMEISTER to find the fine-grained alignments or High-scoring Segment Pairs). We must stress out that the CHROMEISTER method is intended for detecting large synteny blocks and evolutionary rearrangements in a fast fashion using as less resources as possible and therefore it is not aimed (on its own) to detect genes and moreover, its inclusion in the tables would be unfair for the other software methods since the number of genes reported would have to calculated as those covered by the detected synteny blocks (which are large and therefore would report much more genes).

Table 2 shows the percentage of genes detected divided by the total number of genes present in the sequence. Notice that we only used fragments detected in the forward strand and therefore only accounted for genes that are not in the complementary strand (particularly to simplify the additional programs that were developed to check this and to reduce execution times by half, approximately).

We performed the experiment with the following sequences: *Mycoplasma hyopneumoniae 232* and *Mycoplasma hyopneumoniae 7422*, *Escherichia coli IAI39* and *Escherichia coli O157*, and lastly two chromosomes, namely *Canis familiaris* chr. 28 and *Bos taurus* chr. 26. All sequences and their annotations were downloaded from the NCBI database.

Method	*Mycoplasma hyop.*	*Escherichia coli*	Chromosomes
Gecko	322 / 352 (91%)<br>316 / 367 (86%)	1763 / 2377 (74%)<br>1819 / 2673 (68%)	197 / 339 (58%)<br>154 / 313 (49%)
Nucmer	226 / 352 (64%)<br>228 / 367 (62%)	1342 / 2377 (56%)<br>1352 / 2673 (50%)	173 / 339 (51%)<br>139 / 313 (44%)
Chromeister pipeline	217 / 352 (61%)<br>218 / 367 (59%)	1072 / 2377 (45%)<br>1032 / 2673 (38%)	172 / 339 (50%)<br>126 / 313 (40%)

Table 3. Number of genes detected by method and dataset in the comparison. Each cell shows two numbers, namely the amount of genes detected in the forward strand of the query sequence divided by the total number of genes in such strand and sequence, and the second number is likewise for the reference sequence.

The differences that can be seen between the CHROMEISTER pipeline and the rest of the methods is mostly due to the fact that CHROMEISTER filters repetitions out (and therefore a significant percentage of genes). To compensate for this effect, we have re-run the experiment but removing all fragments that had an overlapping of at least 60% in length, as before in the experiment from Table 3. The results are seen in Table 4.

Method	*Mycoplasma hyop.*	*Escherichia coli*	Chromosomes
Gecko	263 / 352 (74%)<br>282 / 367 (76%)	1056 / 2377 (44%)<br>907 / 2673 (33%)	190 / 339 (56%)<br>144 / 313 (46%)
Nucmer	122 / 352 (34%)<br>126 / 367 (34%)	1080 / 2377 (45%)<br>1046 / 2673 (39%)	162 / 339 (47%)<br>123 / 313 (39%)
Chromeister pipeline	209 / 352 (59%)<br>218 / 367 (59%)	989 / 2377 (41%)<br>956 / 2673 (35%)	171 / 339 (50%)<br>126 / 313 (40%)

Table 4. Re-run experiment without overlapping regions to detect the number of genes by method and dataset. Each cell shows two numbers, namely the amount of genes detected in the forward strand of the query sequence divided by the total number of genes in such strand and sequence, and the second number is likewise for the reference sequence.

We can see that results are much closer altogether and even in some cases, the pipeline outperforms NUCMER. Notice also that the number of genes in the second *Escherichia coli* sequence for GECKO is a 2% lower compared to the pipeline, which seems a contradiction. However, it is due to the high number of repetitions in *Escherichia coli* that are found by GECKO but not by the pipeline, which eventually get discarded in the filtering process. This is, in fact, an imperfection of the devised method to remove repeated regions, since it also removes repetitions included in the main signal which in the case of the pipeline do not get removed because no repetitions are detected.

Indexation and matching procedure

First of all we would like to stress (as stated in the manuscript) that the method is designed to use as little information as possible, and as fast and efficiently as possible in order to produce a quick overview of the comparison of two genomes. Although the method is the combination of several heuristics (inexactitude, uniqueness, subsampling and traversal of local optima), in this section we discuss the matching procedure, which includes indexing by both inexactitude and uniqueness. See Figure 1 for an example.

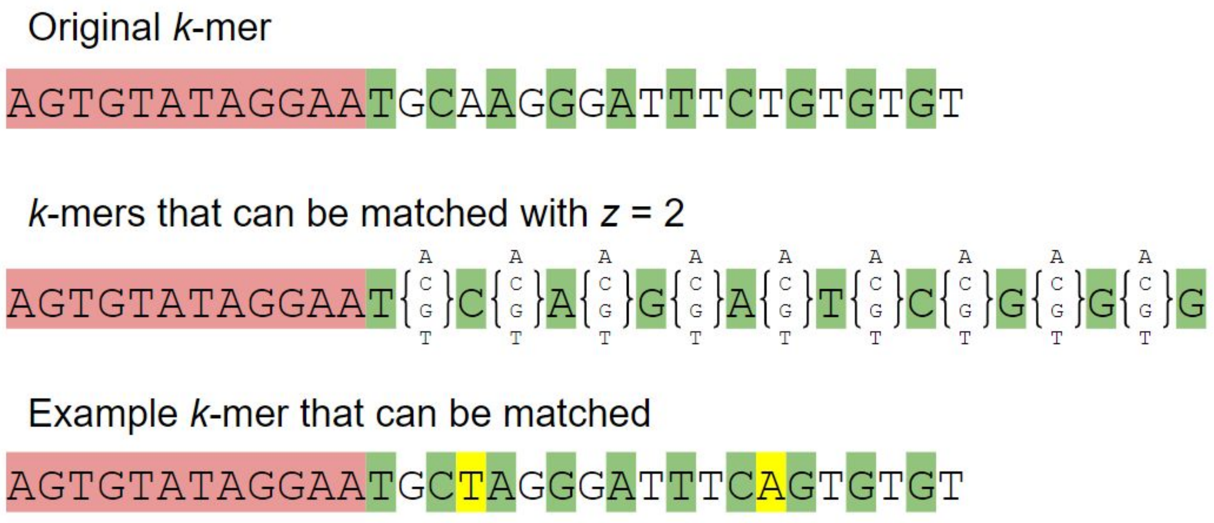

Figure 1. Indexation of *k*-mers using the inexact method with z = 2. The original *k*-mer (shown in the top) is composed of two parts (1) in red, the fixed prefix used to map the *k*-mer to a hash table (key) and (2) in green, the nucleotides used to compute its hash (value). The second part (bottom) shows which *k*-mers can be matched (and therefore converted into hits if uniqueness is satisfied) to the original *k*-mer. The third part shows an example *k*-mer that can be matched, with nucleotides differing from the original one shown in yellow.

In short, the matching procedure is aimed at reducing the search space by allowing to match *k*-mers to a representative *k*-mer of its class (clustering by inexactitude) and then using only the unique prototypes of each class (uniqueness). Freely speaking, it can be considered as an on-the-fly clustering where only centroids are used.

False positives and false negatives due to inexactness

From Figure 1 we can see how the indexing is performed. A prefix (in red) is used to map the *k*-mer key to a hash table whereas the individual nucleotides (in green) are used to calculate the hash value. Notice that this approach enables long *k*-mers (which avoids a large noise to signal ratio) to be matched with other *k*-mers that include single point mutations or have suffered from sequencing errors. This also includes cases where remote sequences are being compared who have suffered from evolution throughout millions of years.

Regarding error rates, we can consider two cases: (1) false negatives and (2) false positives. In one hand (1), a false negative corresponds to not matching two equal *k*-mers. This case is not possible since two equal *k*-mers will have two equal hash values.

On the other hand (2), a false positive would correspond to matching two different *k*-mers. Strictly speaking, the method creates false positives to account for mutations and other evolutionary events. We have performed an experiment addressing the confusion matrix rates, which is shown in the next sections.

False positives and false negatives due to uniqueness

The condition of uniqueness is very restrictive. It is important to keep in mind that it was designed in such a way that the algorithm could run with nearly no memory requirements and boosting performance while producing a fast previsualization. In short, the uniqueness condition removes the problem of handling collisions, balancing trees, etc., i.e. it reduces the problem to a single, key-value hash table. These computational benefits come, however, at the expense of a decrease in sensitivity. Particularly, due to uniqueness, false positives (matching different *k*-mers) are not affected (since their matching is only due to inexactness), however these can be turned into false negatives as explained next, and therefore removed. On the other hand, a large deal of false negatives (not matching equal *k*-mers) will be generated since any *k*-mer that is present twice is considered as a repetition (therefore not being matched with any other *k*-mer to form a hit).

In any case, we performed an experiment to objectively measure how many hits are lost due to uniqueness. We employed the sequences from previous experiments, namely *Mycoplasma hyopneumoniae 232* and *Mycoplasma hyopneumoniae 7422*, *Escherichia coli IAI39* and *Escherichia coli O157*, and included two larger chromosomes, *Homo sapiens* chr X and *Mus musculus* chr X, and disabled inexact matching to only account for unique hits.

Method	*Mycoplasma hyop.*	*Escherichia coli*	*Homo sap. vs. Mus mu.*
All hits	417,633	1,802,818	15,691,528
Unique hits	14,440	57,704	15,152

Table 5. Hits detected using traditional *k*-mer matching and those unique for each of the three datasets.

As can be observed from Table 5, the number of unique hits is not correlated to the number of total hits. Moreover, we calculated the percentage of final fragments that included a unique hit by extracting the aligned fragments from GECKO. Then we removed all repeating regions that had at least 60% of overlap as in previous exercises to account only for the main signal. Table 6 shows the results.

Method	*Mycoplasma hyop.*	*Escherichia coli*	*Homo sap. vs. Mus mu.*
Minimum fragment length	100	200	300
Total aligned frags	324	556	1037
Total frags containing	282 (87%)	543 (97%)	845 (81%)

unique hits			

Table 6. Unique hits contained within aligned fragments. The row “Minimum fragment length” represents the length filter (in base pairs) applied in the comparison. The row “Total aligned frags” shows how many fragments were detected by GECKO. The last row, “Total frags containing unique hits” represents how many of these fragments included the unique hits reported above.

As we can see from Table 6, above 80% of the non-overlapping aligned fragments reported by GECKO (of a certain minimum length) include unique hits. From our perspective, this shows that the uniqueness approach (while dramatically reducing the search space) is able to find a significant proportion of seeds that can later spawn the alignments of the main signal.

Regarding error susceptibility of inexact versus traditional k-mers

First, the inexact design is aimed at enabling long *k*-mers to be matched with other *k*-mers that have suffered some form of mutation (a higher degree of the variable *z* will perform a less restrictive matching and vice versa). However, in order to measure the impact of the inexactness in the matching, we performed an experiment where we took one sequence, generated its *k*-mers, then mutated the sequence (see details below in the description of the experiment) once and re-generated its *k*-mers. Lastly, we matched the initial *k*-mers with those corresponding to the mutated sequence and counted the rates for the confusion matrix. We repeated the experiment with both traditional *k*-mer matching and inexact *k*-mer matching to understand

1. To which degree the traditional method loses sensitivity due to single point mutations compared to the inexact method.
2. To which degree the inexact method matches to other *k*-mers.

For the sake of simplicity, the initial sequence had all *k*-mers different, i.e. each *k*-mer should match only once between the set of *k*-mers from the non-mutated and mutated sequences. Regarding the dataset, the first kilobase of the *Bos taurus* chromosome X was extracted. A mutation probability of 0.01 was applied to each base (resulting in average of 10 mutations per kilobase). We counted the confusion matrix rates as follows:

1. True positive: When a *k*-mer from the non-mutated sequence is matched to its counterpart in the mutated sequence (i.e. they match at the same position).
2. False positive: When a *k*-mer from the non-mutated sequence is matched to any other *k*-mer in the mutated sequence at a different position.
3. True negative: When a *k*-mer from the non-mutated sequence is not matched to a *k*-mer in a different position in the mutated sequence.
4. False negative: When a *k*-mer from the non-mutated sequence is not matched to its counterpart at the same position in the mutated sequence.

Table 7 shows the confusion matrix for the experiment. The number of true negatives is high as it counts for each *k*-mer on the non-mutated sequence if it is matched to any of the other mutated sequence. Regarding true positives, we can see that the inexact method enables to match more *k*-mers than the traditional exact method, as it is not as sensible to mutation. Moreover, the number of false positives is zero in both the traditional method (it can not be otherwise since hashes are bijective) and the inexact one (although this depends on the dataset size, since collisions are possible).

	Traditional exact *k*-mer matching		Inexact *k*-mer matching	
Actual/Predicted	Matched	Non-matched	Matched	Non-matched
Matched	602 (TP)	0 (FP)	785 (TP)	0 (FP)
Non-matched	366 (FN)	937,024 (TN)	183 (FN)	937,024 (TN)

Table 7. Confusion matrix for the traditional versus inexact *k*-mer matching experiment using 1,000 *k*-mers. The row represents the actual class of the *k*-mer whereas the column represents the predicted one.

In short, the experiment depicted shows that inexact *k*-mer matching can reduce the number of false negatives while increasing true positive rates in a scenario where we allow and desire some degree of flexibility in the matching. In cases where we are looking for exact, restrictive matching, then it makes more sense to employ traditional *k*-mer matching. From our perspective, in the context of genome comparison where sequences have diverged several millions of years ago, it is reasonable to employ the inexact method since it not only improves detection but also reduces the computational complexity (less nucleotides need to be hashed is a positive side effect).

Furthermore, we performed a second experiment with nearly 10,000 *k*-mers (9,713 after removal of equal *k*-mers in order to have no false positives) to see the trade off between true positives, false negatives and particularly false positives. The procedure was the same as in the previous experiment, i.e. same mutation rates were applied and no two *k*-mers are equal. Table 8 shows the results of the confusion matrix for the nearly 10,000 *k*-mers.

	Traditional exact *k*-mer matching		Inexact *k*-mer matching	
Actual/Predicted	Matched	Non-matched	Matched	Non-matched
Matched	7,712 (TP)	0 (FP)	8,680 (TP)	205 (FP)
Non-matched	2,001 (FN)	95,197,201 (TN)	1,041 (FN)	95,275,396 (TN)

Table 8. Confusion matrix for the traditional versus inexact *k*-mer matching experiment using 9,713 *k*-mers. The row represents the actual class of the *k*-mer whereas the column represents the predicted one.

As we can see from Table 8, the number of false negatives is reduced by almost 50% in the case of the inexact matching, since it allows to match slightly varying *k*-mers. Moreover, this can also be appreciated in the number of true positives identified while the number of false positives only raises to 205.

Overall, while the inexact approach is able to improve the true positive rate and decrease true negative rate at the expense of a slight increase in false positive rate, we must also point out that these “false positive” matches will rarely have a direct impact in the output, since, as explained before, a sufficient number of matches is required around a region in order to form or cluster signal (and the number of false positives is not only reduced, but their positions will be scattered throughout the comparison).

Pipelined execution

The comparisons were rerun exhaustively with GECKO using the information generated from the previous CHROMEISTER execution. That is, the genomic regions surrounding the detected signals of conversed similarities were compared with GECKO to produce a set of aligned HSPs. Such set of fragments can then be used for post-processing affairs such as in GECKO-MGV. Table 3 shows the execution times for the exhaustive rerun compared to the best execution from either CGALN or NUCMER. Notice that in the previous comparison (main manuscript), the parameters of NUCMER were tweaked to favor it (and for instance, the extension of alignments stage was removed), but for this exhaustive test, parameters are left default in order to produce high quality results.

As can be seen, comparisons B19-C9, HX-MX and BC-BC are slower by few minutes, but still competitive. However, as the search space of the comparison grows (e.g. M2-O1, A1-T1, HC-MC and AC-TC), the pipelined execution outperforms the other methods while providing a higher level of exhaustiveness (see next section) in terms of signal detection and competitive results in terms of gene detection (see section “Sensitivity analysis”). Particularly, the case of M2-O1 shows a time reduction that does not follow the growing trend of the search space. This is due to the time consumed by the pipelined execution being directly proportional to the number of conserved signals detected, for which M2-O1 has the lowest number of similarities. It is also interesting to remark that the pipelined execution required less than 1.5 GB of RAM for any execution, including the full plant genomes. This enables the method to be run even on old computers, tablets or smartphones.

The CSV files containing the sets of HSPs ready-to-run on the GECKO-MGV server[1] are available from the Supplementary Material under section “Pipelined execution”. Nonetheless, the pipelined execution can be easily adapted to work with any other comparison software, such as BLAST or BOWTIE.

---

[1] https://pistacho.ac.uma.es/

	Best performance<br>CGALN or NUCMER		Pipelined execution<br>CHROMEISTER/GECKO pipeline	
Dataset	Time (m)	RAM (MB)	Time (m)	RAM (MB)
B19-C9	2.36	905	0.89 / 5.16	993 / **101**
HX-MX	5.81	2,221	1.29 / 6.77	**999** / **106**
BC-BC	9.96	7,263	2.42 / 20.01	**1,022** / **115**
M2-O1	10.96	6,677	**2.84** / **2.56**	**1,017 / 116**
A1-T1	2,739.10	15,199	**2.84** / **23.65**	**1,046 / 1,002**
HC-MC	170.66	46,038	**13.77** / **93.88**	**1,046 / 208**
AC-TC	>6,000.00	53,500	**15.96** / **212.35**	**1,046 / 1,314**

Table 9. Comparison of the best performance of either CGALN or NUCMER and the exhaustive rerun execution with GECKO.

Notice that as the length of the compared sequences grows, the amount of information compressed per cell in the hits matrix also grows. Therefore, it becomes necessary to increase the resolution of the cell matrix in order to not lose detail due to information compression. For instance, in Table 9, the sequences corresponding to A1-T1, HC-MC and AC-TC, were executed with a double sized hits matrix, thus enabling a higher resolution output and decreasing the number of unnecessary exhaustive comparisons performed by GECKO. The increase in size of the hits matrix does not affect performance, and only impacts the size of the output file in secondary storage.

Signal comparison

The signals produced by each of the methods mentioned are now compared to the proposal in order to validate and assess the quality of the results. That is, the information produced by CHROMEISTER is pipelined into GECKO to obtain a set of HSPs, and these are compared to the HSPs found by the alternative methods. Coverage is measured relative to the query genome used in the comparison. In this case, Bos taurus Chr 19 was compared against Canis familiaris Chr 9. Figure 2 shows the dotplot comparison as generated by GECKO, NUCMER, CGALN and the pipeline. Notice that HSPs were filtered with a minimum of 300 base pairs and 70% identity.

As can be observed, consensus is to be found among the different methods. These results can also be contrasted with public repositories, such as NARCISSE or CoGe.

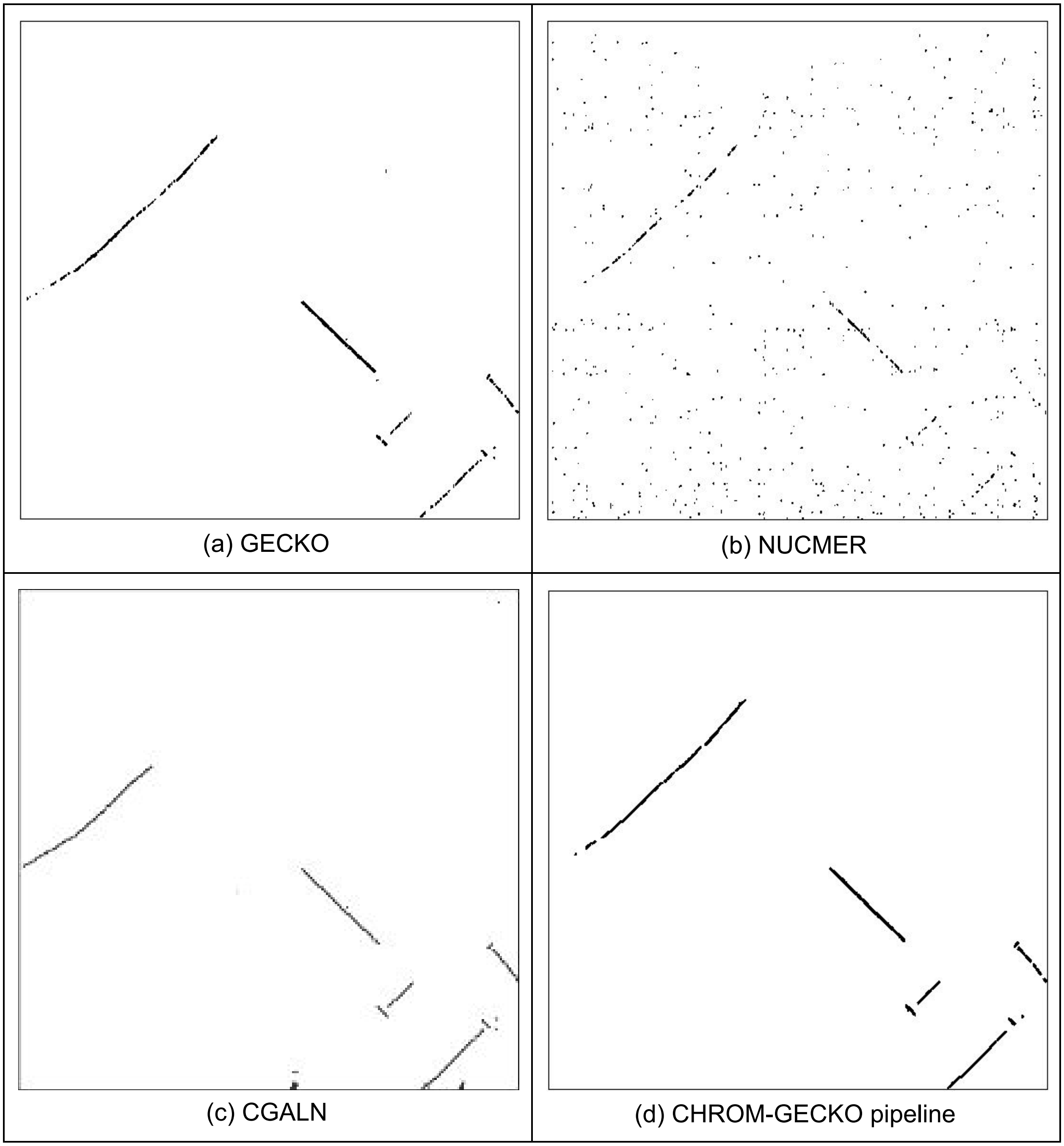

Figure 2. Visual comparison of the coverage obtained from GECKO, NUCMER, CGALN and the CHROMEISTER-GECKO pipeline. Minimum thresholds were set at 300 bp and 70% identity.

As can be observed in Figure 2, mostly all methods agree on the same visual result. However, NUCMER detects isolated and single HSPs throughout the comparison, even so when all thresholds are set equally.

Table 10 shows the coverage values for the comparisons depicted. The total alignment length is normalized in respect to the sequence length of the query (in this case, Bos taurus Chr 19).

Notice that the coverage appears to be low compared to the representation in the dotplots; this is due to the existing separation between HSPs, which can not be observed in thumbnail previews. As seen in Table 10, the signal percentage for the pipeline was superior to that of NUCMER and CGALN, and highly similar to that of the full GECKO execution, which is considered the reference as it provides the longest, high-quality and verifiable set of alignments.

Method	Total alignment length (bp)	Coverage	Signal percentage
GECKO	1,592,140	2.48%	**100%**
NUCMER	620,787	0.96%	38%
CGALN	369,071	0.57%	23%
MEGABLAST*	705,900	1.10%	44%
Pipeline CHROM-GECKO	1,221,658	1.90%	**76%**

Table 10. Comparison of the quality of the signal detected among the different methods. For each method, the total number of base pairs aligned at a minimum of 70% identity and of length above 300 nucleotides is reported. The coverage is computed relative to the size of the query genome. The signal percentage column is computed as the ratio between the coverage from GECKO and the rest of the methods. *MEGABLAST is included as reference without dotplot.

Infrastructure

All the comparisons and executions were performed on our local server at the University of Malaga with 2x 8-core Intel Broadwell-EP E5-2620V4 processor at 2.1 Ghz, 64 GB of RAM and 2TB of local storage at 7,200 rpm. The version of GCC and g++ used to compile all binaries was 6.3.0. The plotting was performed with either R version 3.3 or Gnuplot 4.2 patchlevel 2.

Three computational limits were set to avoid infinite computing times, namely a 100 hours of CPU time, 64GB of RAM and 300GB of hard disk drive.

Detected Large-Scale Genome Rearrangements in the synteny map

The algorithm devised for detecting LSGRs is available at the github of the project (find it here: https://github.com/estebanpw/chromeister). It is a parametric algorithm that enables a variable degree of edge detection, and must be adjusted depending on the comparison being performed (e.g. small sequences, chromosomes or full sized genomes). For the similarity map experiment we employed the following parameters:

Parameter	Value
WSIZE	7
TH	5

penalty	15
sidePenalty	3
reward	6

Table 11. Parameters employed in the LSGR detection.

The amount of evolutionary events detected for each comparison with *Homo sapiens* is displayed in Table 12. Notice, however, that the algorithm employed counts synteny blocks, *i.e.* more synteny blocks imply more evolutionary events (as long as there still is conserved similarity) since they have to be broken apart to separate into more synteny blocks.

Species	Number of LSGR compared with *Homo sapiens*
Pan troglodytes	8
Pan paniscus	7
Gorilla gorilla	6
Pongo abelii	12
Nomascus leucogenys	64
Papio anubis	36
Macaca mulatta	36
Macaca fascicularis	31
Chlorocebus sabaeus	44
Callithrix jacchus	47
Microcebus murinus	50

Table 12. Number of LSGR found in each comparison with the *Homo sapiens* genome as reference.

Correlation between LSGRs and the scoring distance

The number of LSGRs and the scoring distance was calculated for all primate species in respect to *Homo sapiens*. Figure 3 shows the visual correlation between both functions. The Pearson's correlation coefficient was 0.72, which indicates that a positive, relatively strong correlation exists. Notice that the score of Homo Sapiens against itself is not 0 due to the huge islands of unidentified nucleotides (see Table 13). These are not computed by CHROMEISTER and can include up to 30,000,000 nucleotides which can impact the overall score.

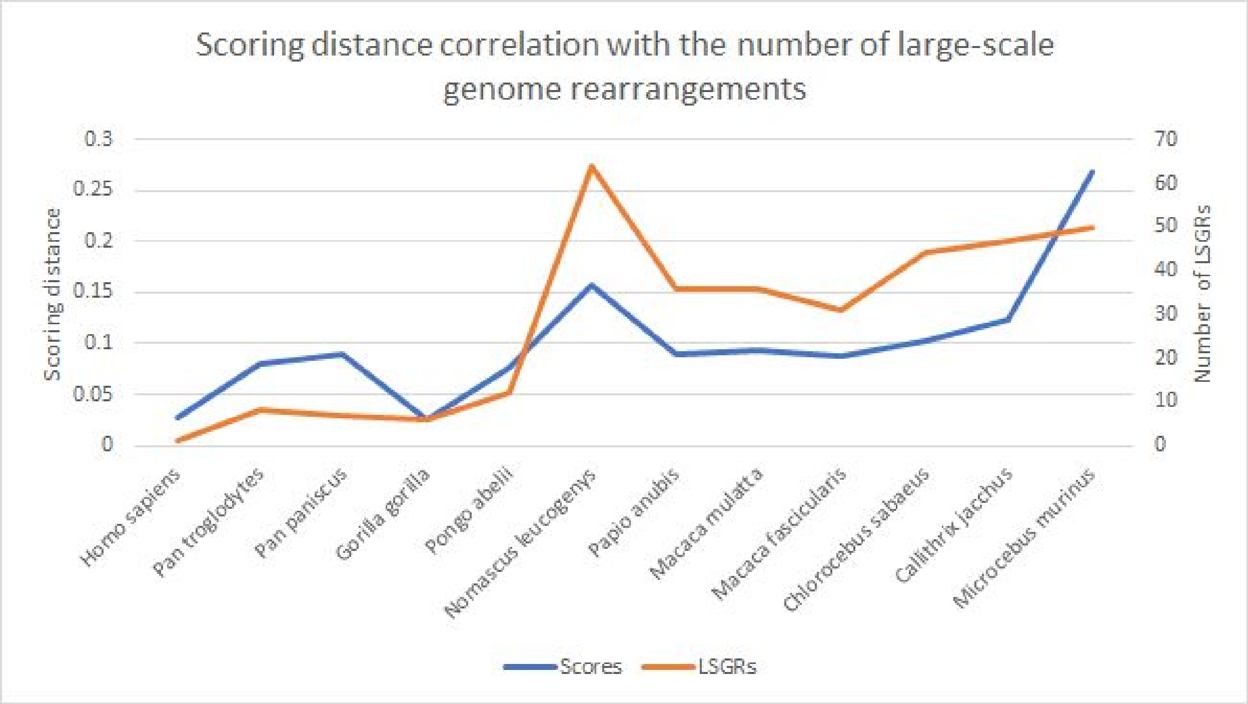

Figure 3. Scoring distance (blue line) versus the number of LSGRs (orange line) for all primate species considered in the experiment, in regard to Homo Sapiens.

Region start	Region end	Length in N's
121485489	142535489	21050000
339795834	340845834	1050000
341576902	344576902	3000000
582955013	585955013	3000000
740132761	743132761	3000000
928032616	931032616	3000000
1121322456	1124422456	3100000
1291711743	1294711743	3000000
1436635017	1439635017	3000000
1586477886	1604627886	18150000
1719528630	1722728630	3200000
1867502704	1870602704	3100000

1985771766	1988771766	3000000
2084767024	2103787024	19020000
2199936959	2218936959	19000000
2307286556	2327286556	20000000
2445103805	2456203805	11100000
2522435819	2525435819	3000000
2596778977	2599878977	3100000
2684077165	2687177165	3100000
2744893991	2747993991	3100000
2781599998	2791011191	9411193
2792788127	2795938127	3150000
2829729949	2845779949	16050000
2939616582	2942716582	3100000
3036305190	3038954710	2649520
3046409743	3049409743	3000000
3065124551	3095124551	30000000

Table 13. Coordinates of regions containing only N's (unidentified nucleotides) in the *Homo sapiens* genome. Only those above 1,000,000 nucleotides long are displayed.

Block tracing

The blocks traced appearing in Figure 4 in the main manuscript are obtained from Tables 14 and 15. The proposed method identifies synteny blocks, providing coordinates for each block. Then, if overlapping blocks are found, they are considered related blocks. As can be seen, the first row in both tables are related to larger blocks (in Figure 4 they would represent a block filling almost the whole chromosome), however, just a fragment of it has been selected, as it is the section that is shared with all the other comparisons. Coordinates are scaled from 1 to 1,000 in respect to their original size. Notice that each comparison employs in the x-axis the sequence that originally was in the y-axis of the previous one, hence allowing to follow blocks from the root to the leaves.

xStart	yStart	xEnd	yEnd	Length (approx.)	Comparison

9	14	1000	1000	984	HS6-PA6
254	4	404	144	139	PA6-MM13
54	279	169	344	65	MM13-EQ20
664	234	790	14	219	EQ20-BT23

Table 14. Scaled coordinates for the blue block displayed in Figure 4 in the main manuscript.

xStart	yStart	xEnd	yEnd	Length (approx.)	Comparison
9	14	1000	1000	984	HS6-PA6
279	184	319	239	54	PA6-MM17
524	279	589	319	40	MM17-EQ20
129	514	479	819	304	EQ20-BT23

Table 15. Scaled coordinates for the yellow block displayed in Figure 4 in the main manuscript.

Parallel execution

The parallelisation of CHROMEISTER is immediate at a task-level paradigm. That is, since the computation of each comparison takes a small amount of time, data-level parallelisation is not as efficient as a coarse-grained task-level paradigm (mostly due to the former requiring synchronisation techniques that clog up computation and add overhead). Therefore, the current implementation of CHROMEISTER includes a script for all versus all comparisons which can be run in parallel. The script is called onto one or two folders, and it will compare the sequences within or between the folders in order. Each time the script is called (with the same command line) another process will be spawned using one core and comparing the next sequences in the order list.

The parallelisation level is done at operating system level, i.e. each process checks that the current two sequences to be compared are not already being compared by another process.

Distribution of hits

Let $A = \{A_1, A_2, \ldots A_n\}$ and $B = \{B_1, B_2, \ldots B_m\}$ be two sequences with independent and identically distributed letters taken from the finite alphabet $S = \{A, C, G, T\}$. Under the assumption that the probability of each letter in S follows an uniform distribution such that:

$$P(A_i = x) = P(B_i = x) = F_i = 1/4,\ i \in S$$

and

$$F_a + F_c + F_g + F_t = 1$$

Then the number of random matches that occur between the two sequences A and B follow a binomial distribution of mean $n * p$ and variance $n * p * (1 - p)$ where n is the number of events (each comparison that can produce a match or not) and p is the probability of each match. The variables n and p will take its value depending on the choice of the k-mer size and the length of the query and the database. For instance, in the case of one query sequence and one database sequence of lengths l_1 and l_2 (respectively), and a choice of k-mer size being k, there would be $(l_1 - k + 1) * (l_2 - k + 1)$ comparisons of k-mers, each of having a probability of being equal of ¼ to the power of k. To accomplish this, the query is considered as a single long sequence, since it is of no use to distinguish between query sequences. Thus the number of k-mers present in a query of Q sequences is the union of the k-mers from each sequence Q_i . On the other hand, each sequence in the database is considered independently, therefore producing S binomial distributions, result of comparing the hits between the whole query as one long sequence and each individual sequence S_i in the database. In particular, for a query of Q sequences each of length Q_i, and a database composed of S sequences, each of them of length S_i, and a k-mer choice of k, the number of k-mer comparisons between the query and each one of the databases will be:

$$n_k = (l_1 - k + 1) * 2 * (l_2 - k + 1)$$

Notice that the number of k-mers in the reference sequence has to be multiplied by two to consider the reverse complementary strand. Therefore, the resulting binomial distribution is modelled as:

$$Bi(n_k, \tfrac{1}{4}^k)$$

Impact of inexact indexing

The impact of the order of z in the indexing function is analyzed. That is, the sequences depicted in the previous section were compared while varying the z value, and the number of unique hits was recorded. The range used for the z value is the sequence $1, 2, 3, 4, 5, 6, 7, 10, 16, 32$ (missing values such as 8 and 9 produce the same results under function $f(x)^z$ as their listed previous integer). Figure 4 shows the number of unique and inexact hits found as a function of z. It can be seen that the sequence comparisons (sorted by ascending search space in the legend) shows a right-skewed distribution that approximates a normal distribution with the growth of the search space. Furthermore, the effect of the z value can be divided into four stages that repeat themselves in each comparison, namely:

1. The initial mild growth is indicative that few inexactitudes are being allowed in the matching of hits, and therefore few and closely related new inexact hits are matched.
2. The steep growth indicates that the allowance degree of inexactitude in the matching is producing a large number of new unique hit matches but with less similarity.

3. The steep decay is indicative for the inexactitude constraints saturating the matching of hits; that is, highly different k-mers are being matched as equal, thus reducing the uniqueness of hits (because almost everything tends to produce more than one hit).
4. The ending mild decay appears when the number of allowed inexactitudes completely saturates the matching of hits (everything matches to everything with no significant similarity).

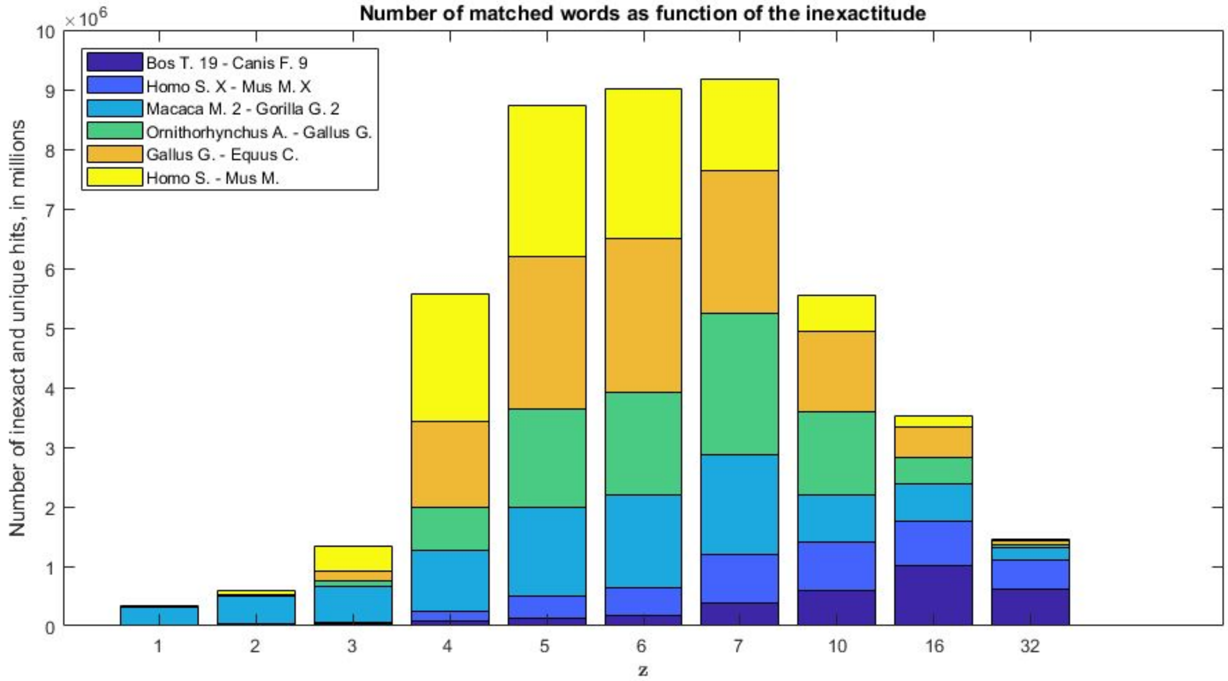

Figure 4. Impact of $f(x)^z$ in the number of unique inexact hits. The x-axis shows the z value, whereas the y-axis shows the number of unique inexact hits found in each of the comparisons. Sequences are sorted from smaller to larger in the legend.

By observing Figure 4, we might conclude empirically that the optimal number of mismatches that should be allowed per sequence comparison has a natural distribution that depends on both input sequences (apparently on the search space). Therefore, with the aim of producing high-quality indexing of unique, inexact hits, the z value should be modified according to the lengths of the sequence, and particularly avoiding the saturation of the hits indexing. The same experiment depicted here is also reproduced for plant genomes (see "Plant genomes and uniqueness" below). Notice, however, that the degree of z is flexible and will still produce high-quality results (all following experiments described in this manuscript were run with $z = 4$).

Plant genomes and uniqueness

The impact of the order of z in the indexing function was analyzed for mammalian sequences. That is, plant genomes were compared while varying the z value, and the number of unique hits was recorded. The range used for the z value is the sequence $1, 2, 3, 4, 5, 6, 7, 10, 16, 32$ (missing values such as 8 and 9 produce the same results under function $f(x)^z$). Figure 5 shows the

number of unique and inexact hits found as a function of z. The following plant sequences and genomes were employed (1) Brassica oleracea - Brassica rapa, (2) Aegilops tauschii chr. 1 - Triticum aestivum chr. 1, (3) Zea mays - Sorghum bicolor and (4) Aegilops tauschii - Triticum aestivum.

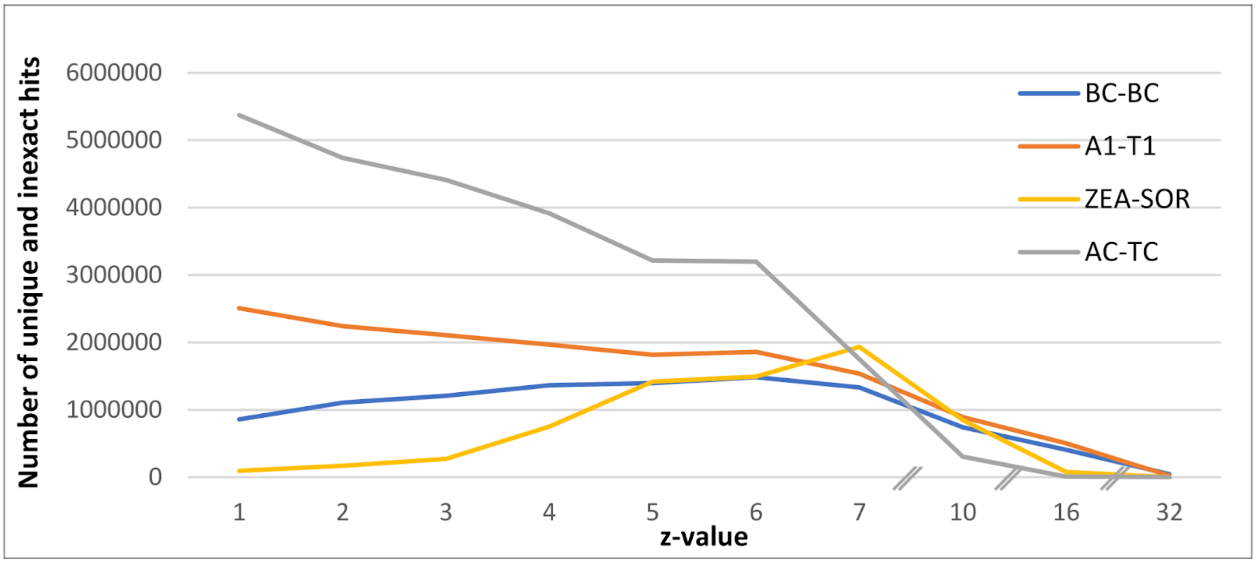

Figure 5. Impact of $f(x)^z$ in the number of unique inexact hits for plant sequences. The x-axis shows the z value, whereas the y-axis shows the number of unique inexact hits found in each of the comparisons. Sequences are sorted from smaller to larger in the legend.

Further visualization

In this section we include the visualization plots for the comparisons that do not appear on the main manuscript. These include M2-O1, BC-BC, HC-MC and AC-TC (see manuscript for more information). Only the previsualization plot generated by CHROMEISTER is shown.

Comparison M2-O1

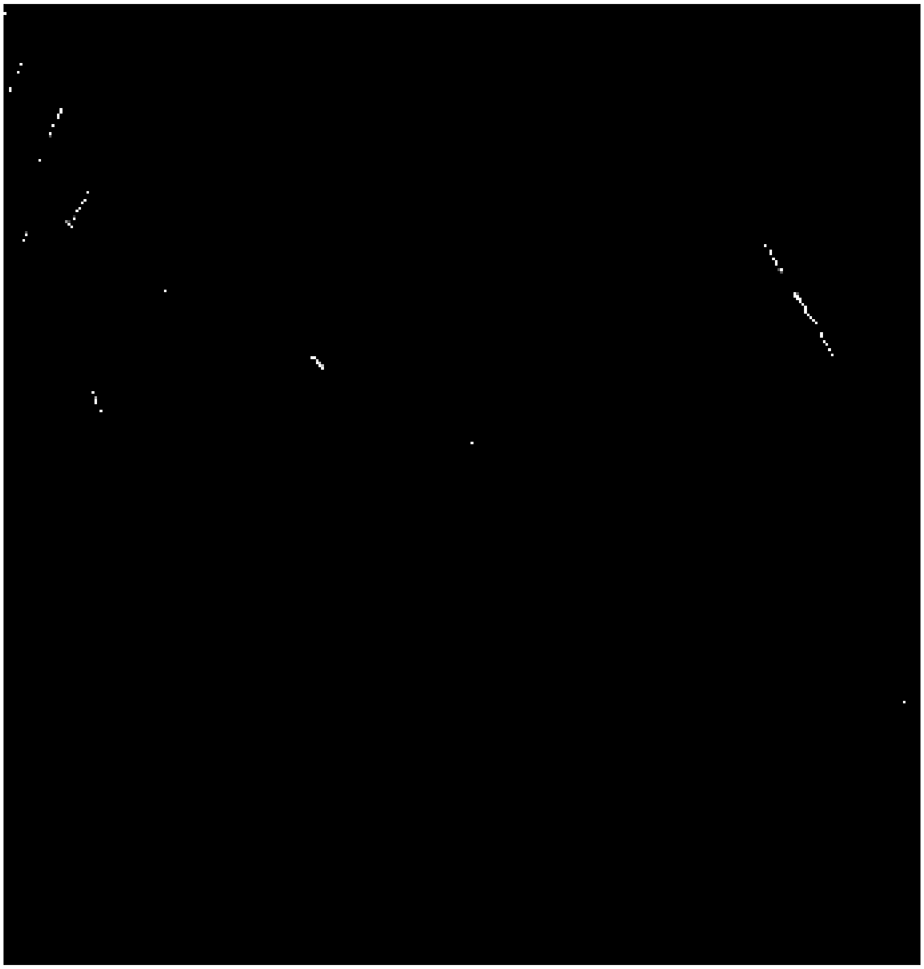

Figure 6. Comparison of *Monodelphis domestica* chr. 2 and Ovis aries chr. 1.

Comparison BC-BC

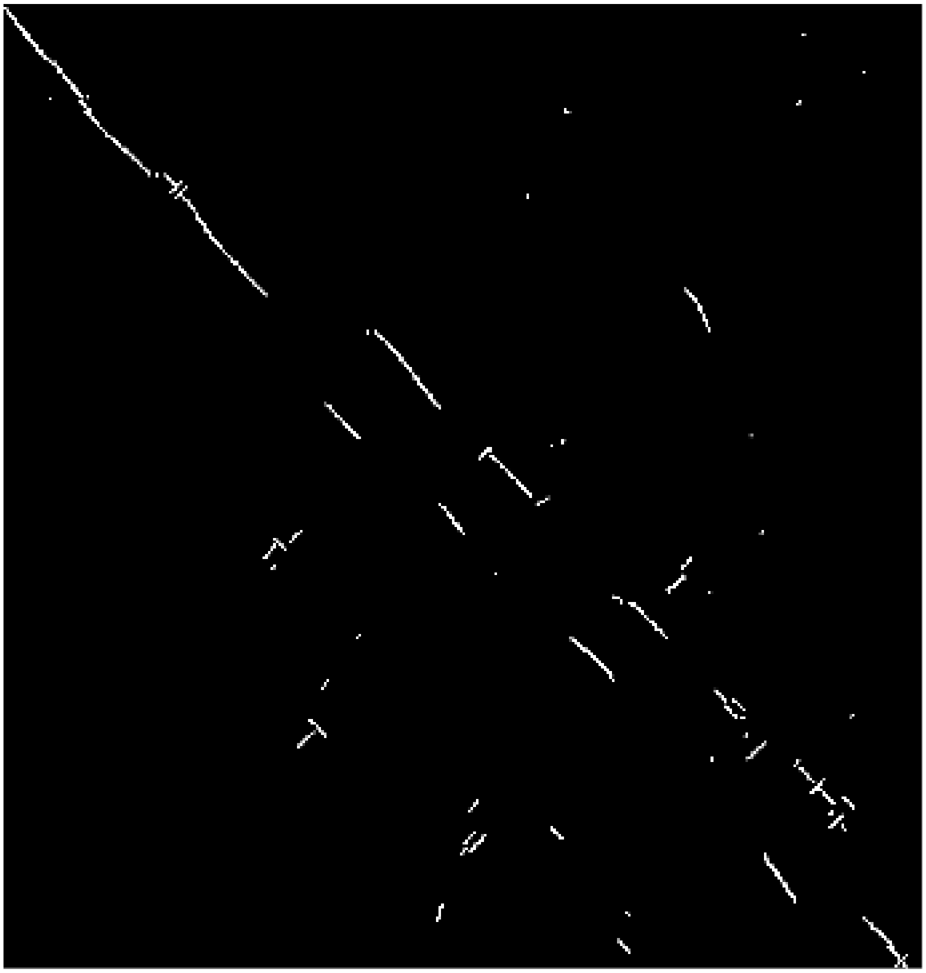

Figure 7. Comparison of *Brassica oleracea* and *Brassica rapa*, all chromosomes in order by size.

Comparison HC-MC

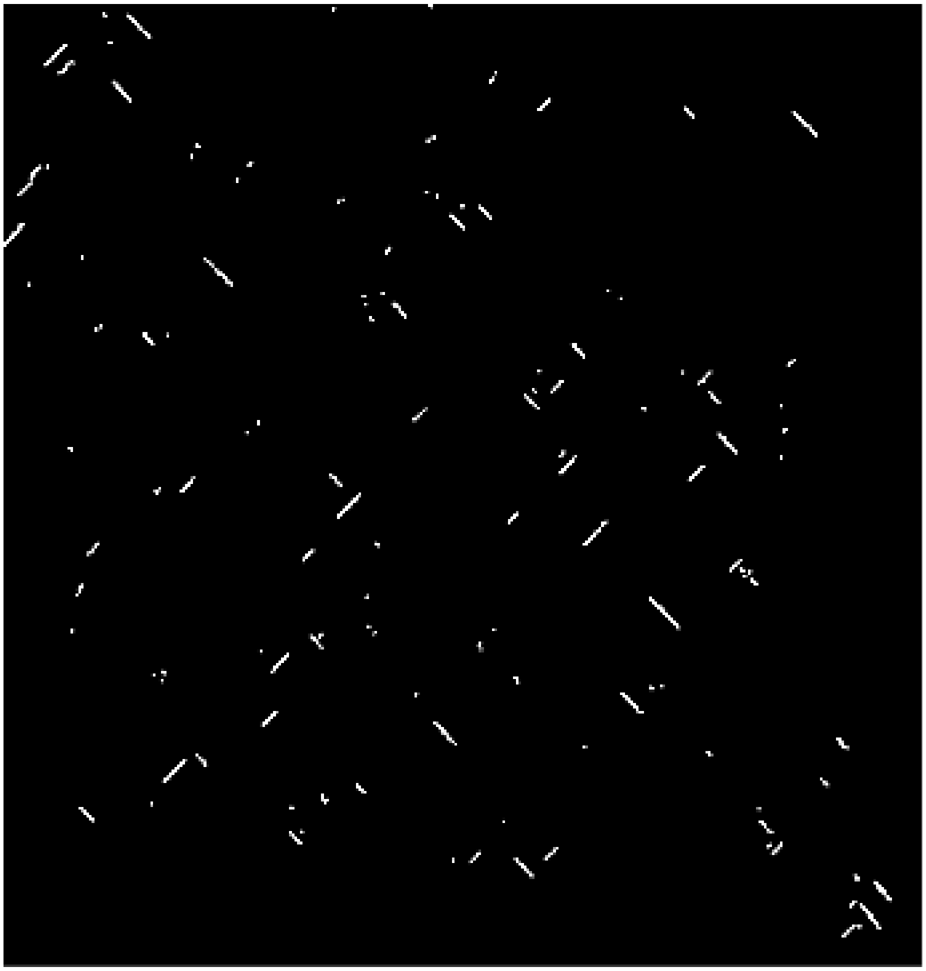

Figure 8. Comparison of *Homo sapiens* and *Mus musculus*, complete genomes built from their chromosomes in sequential order.

Comparison AC-TC

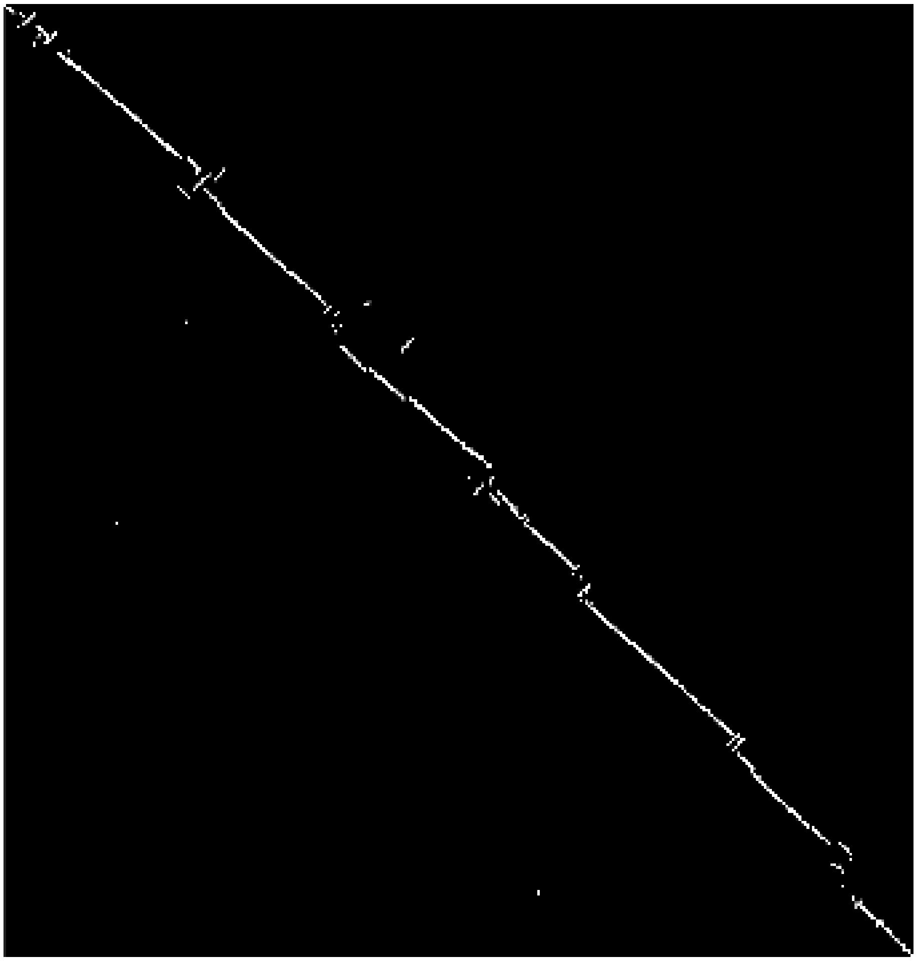

Figure 9. Comparison of *Aegilops tauschii* and *Triticum aestivum*, complete genomes.

Software parameters

Regarding each of the programs:

- CGALN was used in the following way:
  - The maketable program was run with different K-mer sizes such as 18, 19, 20, 30 and 32. But all of them produced a segmentation fault, except for the default size 11.
  - The -r -b flags were set for fast computation.
- NUCMER was used with the following parameters:
  - nucmer -t 1 --noextend --mum -l 32
  - Time included generating the files for plotting and running mummerplot.
- CHROMEISTER was used default. Time included plotting.

NUCMER CPU usage

Despite using the parameter -t 1 to avoid multithreading, NUCMER would utilize more than one core for every test. Therefore, the amount of CPU% used was measured with the time utility and recorded.

Single One vs. One chromosome comparison and software comparison

In this section, we show several comparisons of standard state-of-the-art chromosomes and genomes belonging to mammals and plants. Additionally, CPU time and RAM consumption is measured and compared with CGALN and NUCMER, part of the MUMMER suite.

The results of three comparisons, namely B19-C9, HX-MX and A1-T1 are shown in Figure 10. The correctness of the B19-C9 and HX-MX comparisons are checked against the NARCISSE portal. Notice that the A1-T1 comparison was found nowhere on public repositories, and therefore its inspection was done in respect to NUCMER. The visualization of the additional comparisons (BC-BC, M2-O1, HC-MC and AC-TC) is available at the Supplementary Material under section "Further visualization".

(a) *Bos taurus* Chr 19 *vs. Canis familiaris* Chr 9

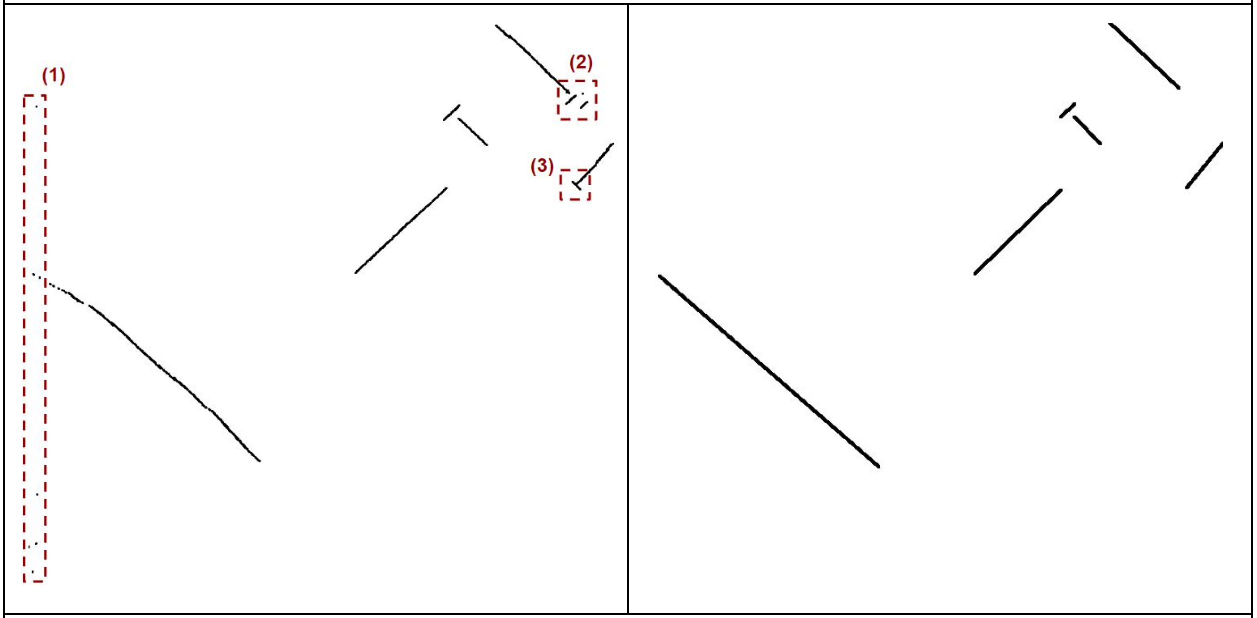

(b) *Homo sapiens* Chr X *vs. Mus musculus* Chr X.

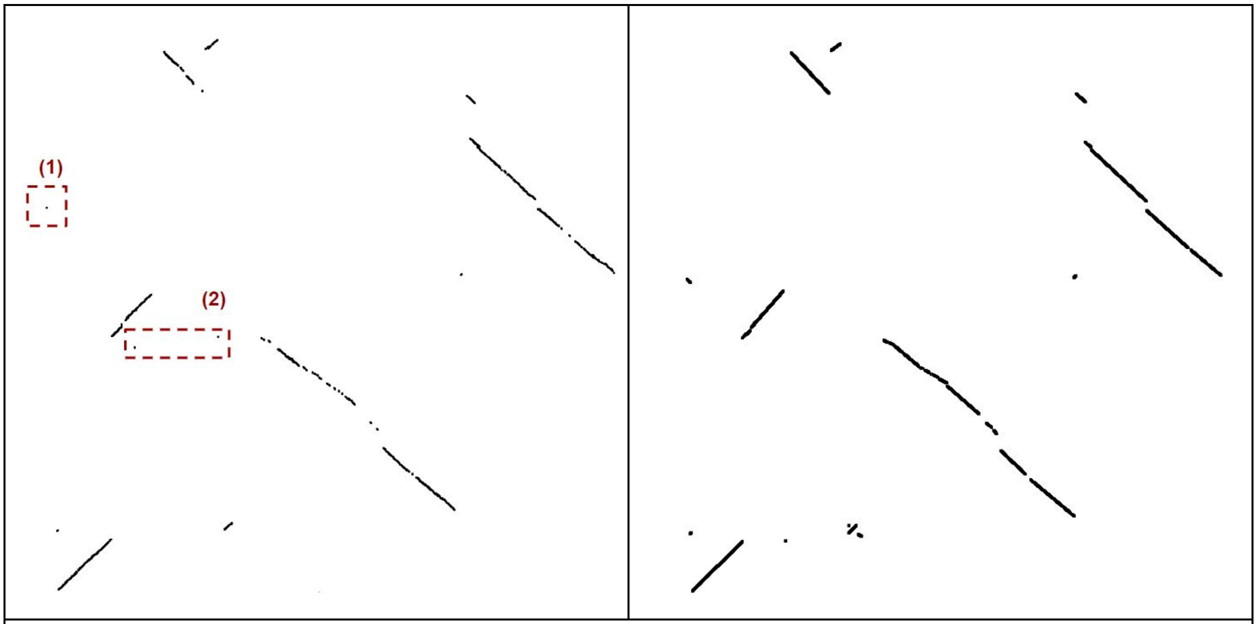

(c) *Aegilops tauschii* assem. Chr 1 *vs. Triticum aestivum* pseudo-Chr 1

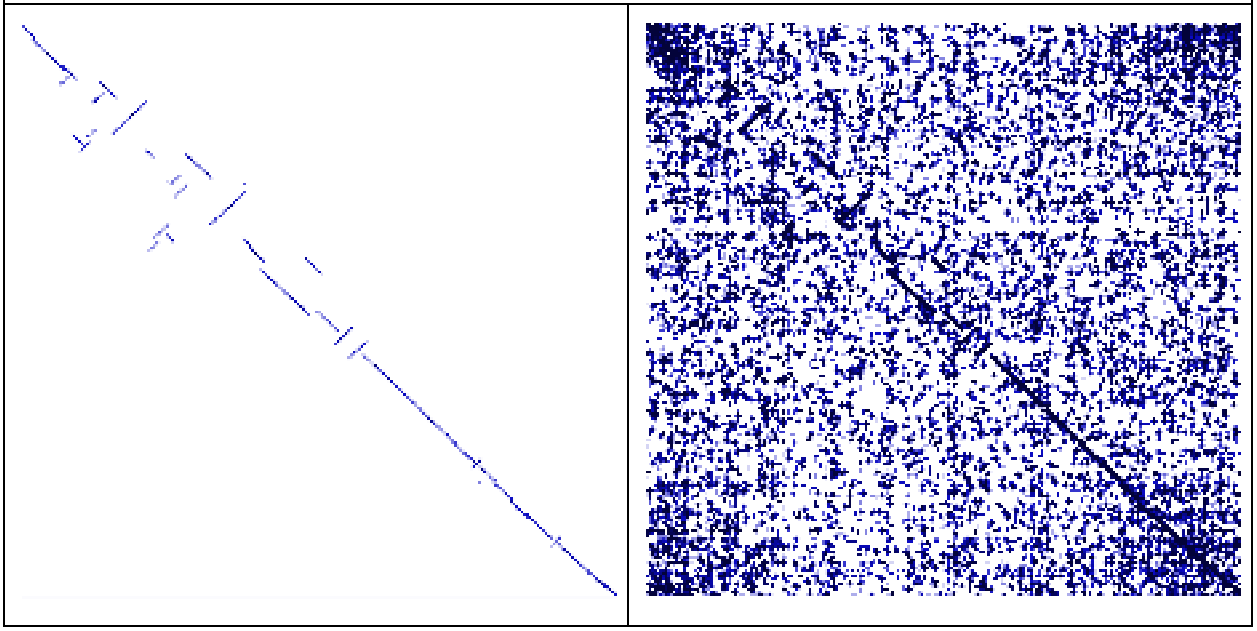

Figure 10. Comparison of the One *vs.* One experiment. The x-axis of each image corresponds to the first sequence in each comparison, whereas the y-axis corresponds to the second sequence. The dotplots on the left are produced by the proposed method, whereas those on the right have been extracted from the NARCISSE platform (except for the last one, which was generated with NUCMER). Differences that are not found in the reference are highlighted in red.

It can be observed from Figure 10 that both (a) and (b) are almost equivalent to the NARCISSE resource. However, notice that the NARCISSE resource has undergone several post-processing refinements and therefore the conserved similarities are observed as much more "cleaner" diagonals than in the case of the proposed method. Still, few differences can be

identified (marked in red). Blocks detected by CHROMEISTER which are undetected by NARCISSE are due to the latter computing the pairwise comparison using gene annotated sets to account for conserved segments. See the Supplementary Material to find more details on these blocks. On the other hand, some conserved segments of lesser size detected by NARCISSE are not detected in CHROMEISTER due to its heuristic nature, i.e. blocks will be detected based on their number of unique hits instead of their gene content, although this is not mutually exclusive (see section "Sensitivity analysis and impact of the heuristic methodologies"

On the other hand, the (c) comparison was contrasted with the result of NUCMER since the comparison did not exist in the considered repository (and which had to be manually filtered, see "NUCMER comparison" in the Supplementary Material). Both methods detect the conserved homologies, although CHROMEISTER removes any repetitive element and therefore only the main syntenies are to be seen, whereas NUCMER shows both repeating regions and homologies, as well as also some noise elements.

Regarding computational times, we set a maximum of 100 hours of computing time to limit execution times. Running times along with the memory ram consumption are shown in Table 16 for NUCMER, CGALN and the proposed method. Only one core was used for each comparison (see "Software parameters" in the Supplementary Material for the list of parameters). The parameters of NUCMER were tweaked to favor it, as it would take more than several thousand minutes on certain datasets with default parameters.

	CGALN		NUCMER		CHROMEISTER	
Dataset	Time (m)	RAM (MB)	Time (m)	RAM (MB)	Time (m)	RAM (MB)
B19-C9	1.63	**755**	1.49 (2.13*)	812	**0.89**	994
HX-MX	5.93	2,032	3.60 (5.14*)	2,221	**1.29**	**998**
BC-BC	141.26	**583**	8.90 (13.61*)	5,814	**2.42**	1,022
M2-O1	17.04	3,618	9.18 (15.14*)	6,436	**2.84**	**1,017**
A1-T1	DNF**	DNF**	7.50 (11.40*)	8,357	**2.84**	**1,022**
HC-MC	DNF**	DNF**	136.18 (168.86*)	46,091	**13.77**	**1,022**
AC-TC	DNF**	DNF**	121.10 (176.80*)	52,021	**15.96**	**1,022**

Table 16. Summarized executions. Each tested program is run against the three single datasets. *NUCMER used more than one core in the computation, the adjusted time is shown in parenthesis, which equals the reported time consumption multiplied by the CPU usage. **The acronym "DNF" is short for "Did Not Finish", and the flag is set when the execution had not finished after 100 hours of computation time.

As can be observed, the proposed method CHROMEISTER is the fastest on every comparison and uses the lowest amount of RAM except for the first dataset B19-C9. CGALN was unable to finish any of the larger comparisons due to time limit. While in the smaller datasets the difference in computation time is not very significative between NUCMER and CHROMEISTER, the gap widens on the large comparisons such as HC-MC and AC-TC, obtaining a speedup of 12x and 11x. Regarding RAM consumption, CHROMEISTER is able to use up to 50 times less than NUCMER in the larger comparisons regardless of the size of the input sequences and therefore making it suitable for its use on any desktop computer.

Proof for new fragments detected between *Bos taurus* 19 and *Canis familiaris* 9

The "Single One vs. One chromosome comparison and software comparison" experiment depicted several similarity blocks that were not identified in the reference, i.e. NARCISSE. In order to ensure that the blocks had indeed similarity, we proceeded to align them using GECKO and loaded the comparison along with the annotation files from GenBank in the MG-Visualizator. Such enables to visualize both the similarity between sequences and whether coding regions are shared.

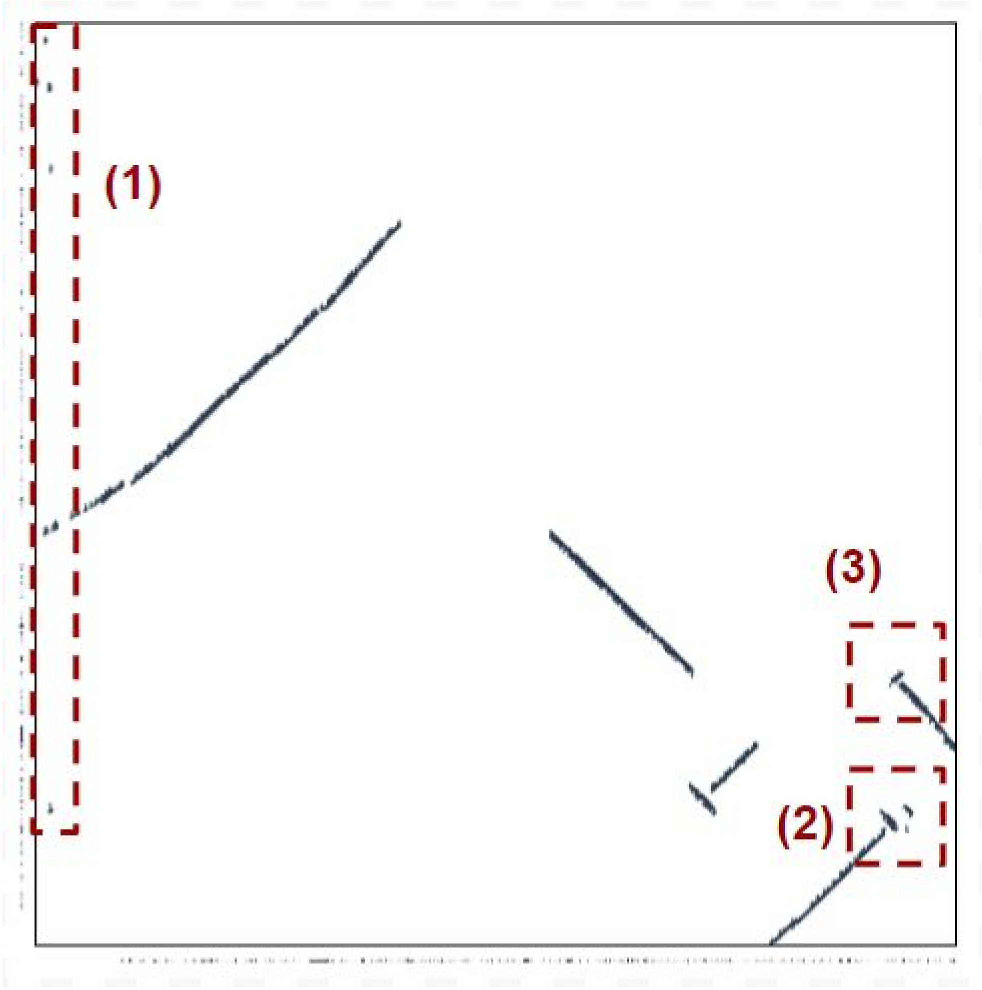

Figure 11. Comparison of *Bos taurus* chromosome 19 and *Canis familiaris* chromosome 9 using the MG-Visualizator. Annotation files were included (scattered points around the axes).

We now check the regions identified by CHROMEISTER, as displayed in red in Figure 11. From this figure we can see that there are no coding regions for the *Bos taurus* chromosome 19, but there are several involved for *Canis familiaris* chromosome 9. This can be seen as well in region 2 in Figure 12.

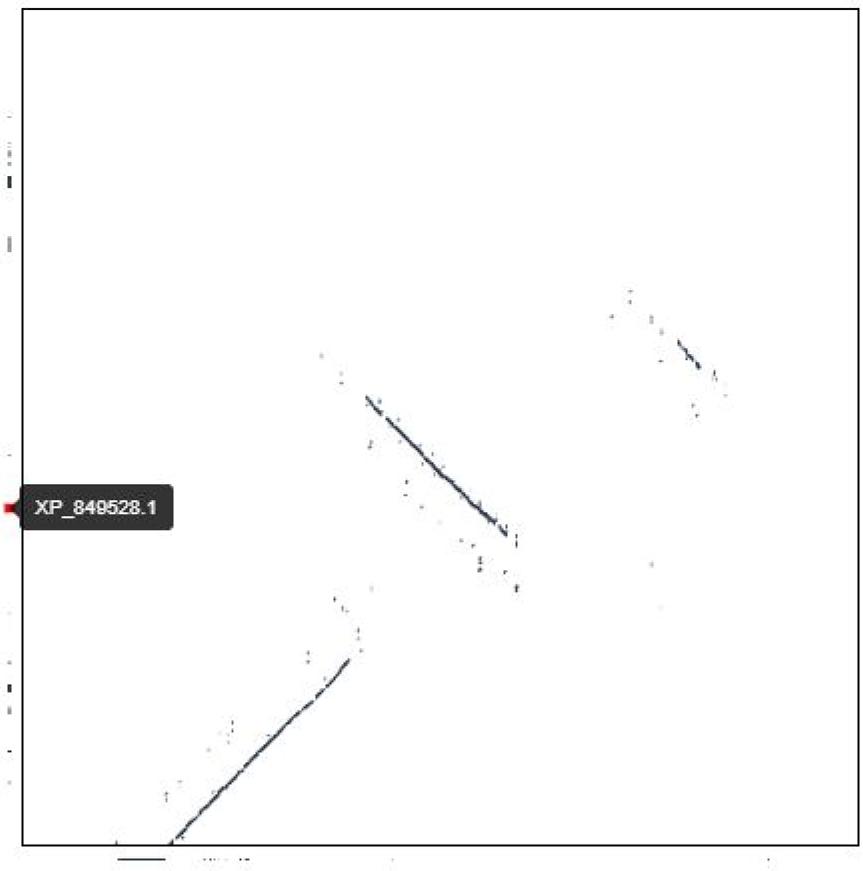

Figure 12. Zoomed region for blocks in part 2.

Notice that in Figure 12, no coding regions exist for sequence in the X-axis but one exists for the Y-axis. Anyway, we can see the two blocks are aligned properly, and that the reason they do not appear on NARCISSE is due to the lack of CDS. The very same scenario can be seen for region 3 in Figure 13.

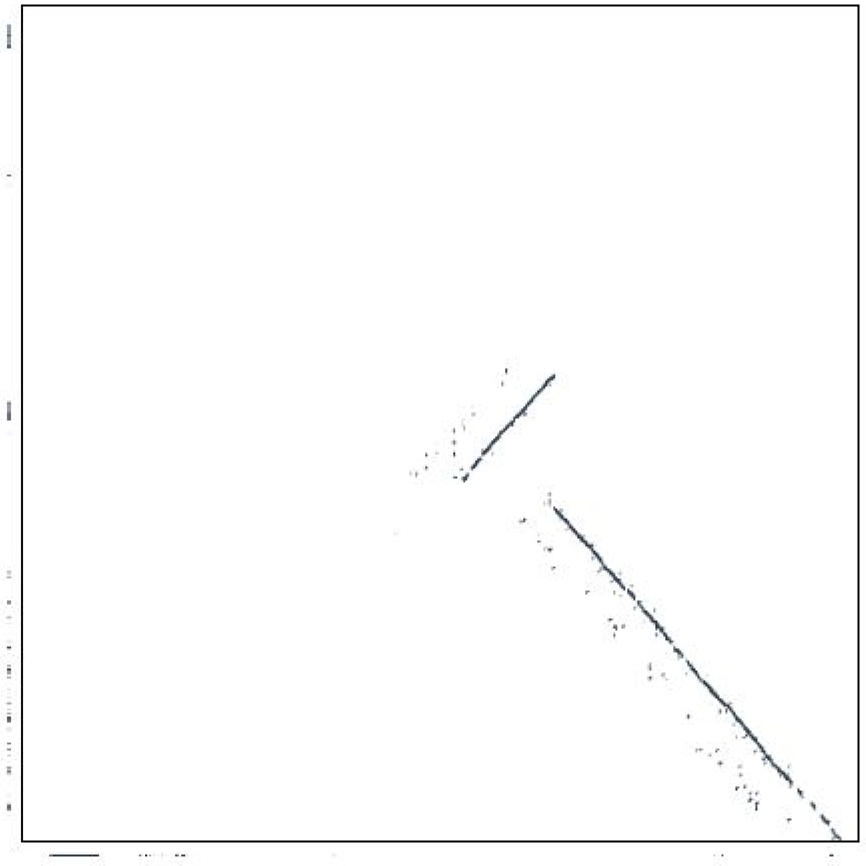

Figure 13. Zoomed region for blocks in part 3.

The block once again exists and aligned between both sequences. However, no coding region is shared between the two.

Proof for new fragments detected between *Homo sapiens* X and *Mus musculus* X.

The “Single One vs. One chromosome comparison and software comparison” experiment depicted several similarity blocks that were not identified in the reference, i.e. NARCISSE. In order to ensure that the blocks had indeed similarity, we proceeded to align them using GECKO and loaded the comparison along with the annotation files from GenBank in the MG-Visualizator. Such enables to visualize both the similarity between sequences and whether coding regions are shared.

Two regions of interest are shown (see Figure 14), aligned with GECKO and zoomed in order to enable visual inspection.

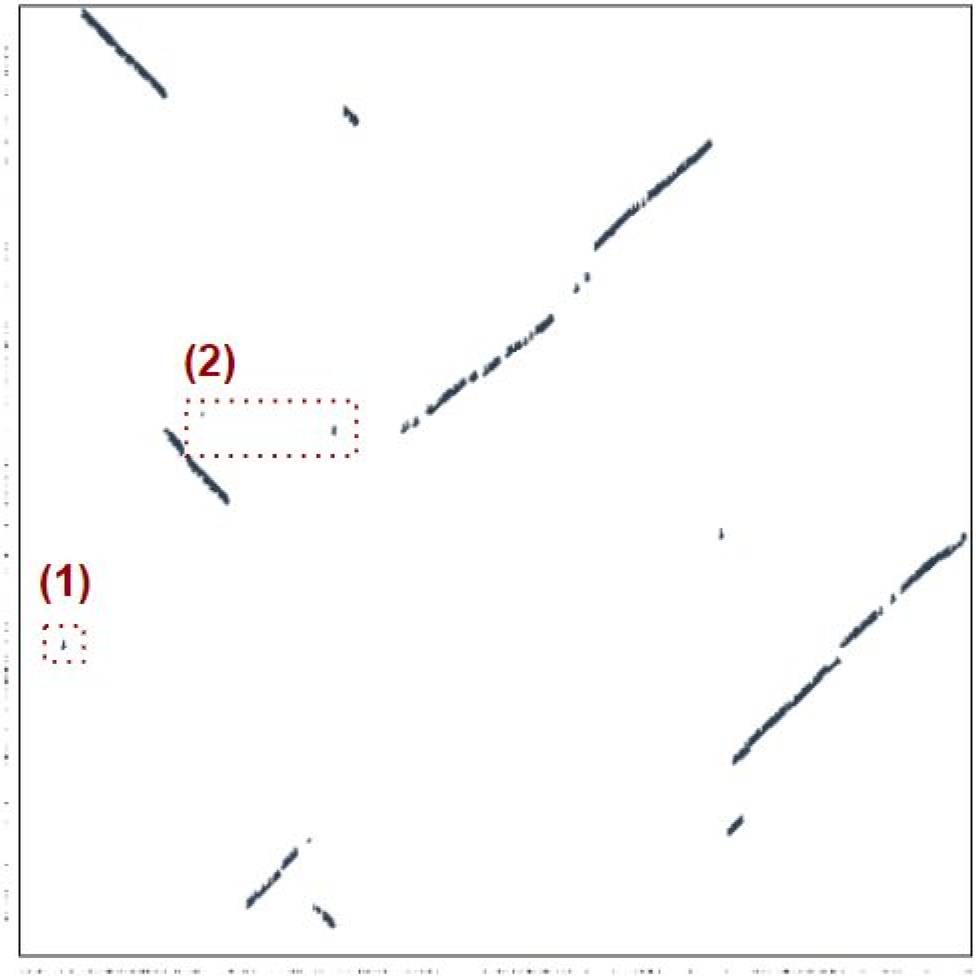

Figure 14. Comparison of *Homo sapiens* chromosome X and *Mus musculus* chromosome X using the MG-Visualizator. Annotation files were included (scattered points around the axes).

We now check the regions identified by CHROMEISTER, as displayed in red in Figure 14. A fragment of length 678 base pairs is detected in the point of interest regarding region 1. Although the fragment is not very large, it proves that the method is consistent with its reported similarities.

The same is observed for region 2, see Figure 15, where the block is 840 base pairs length and Figure 15 where the block has 218 base pairs length. This shows that the length of the block does not necessarily imply that the block will be skipped - it is rather a combination of the uniqueness in its composition.

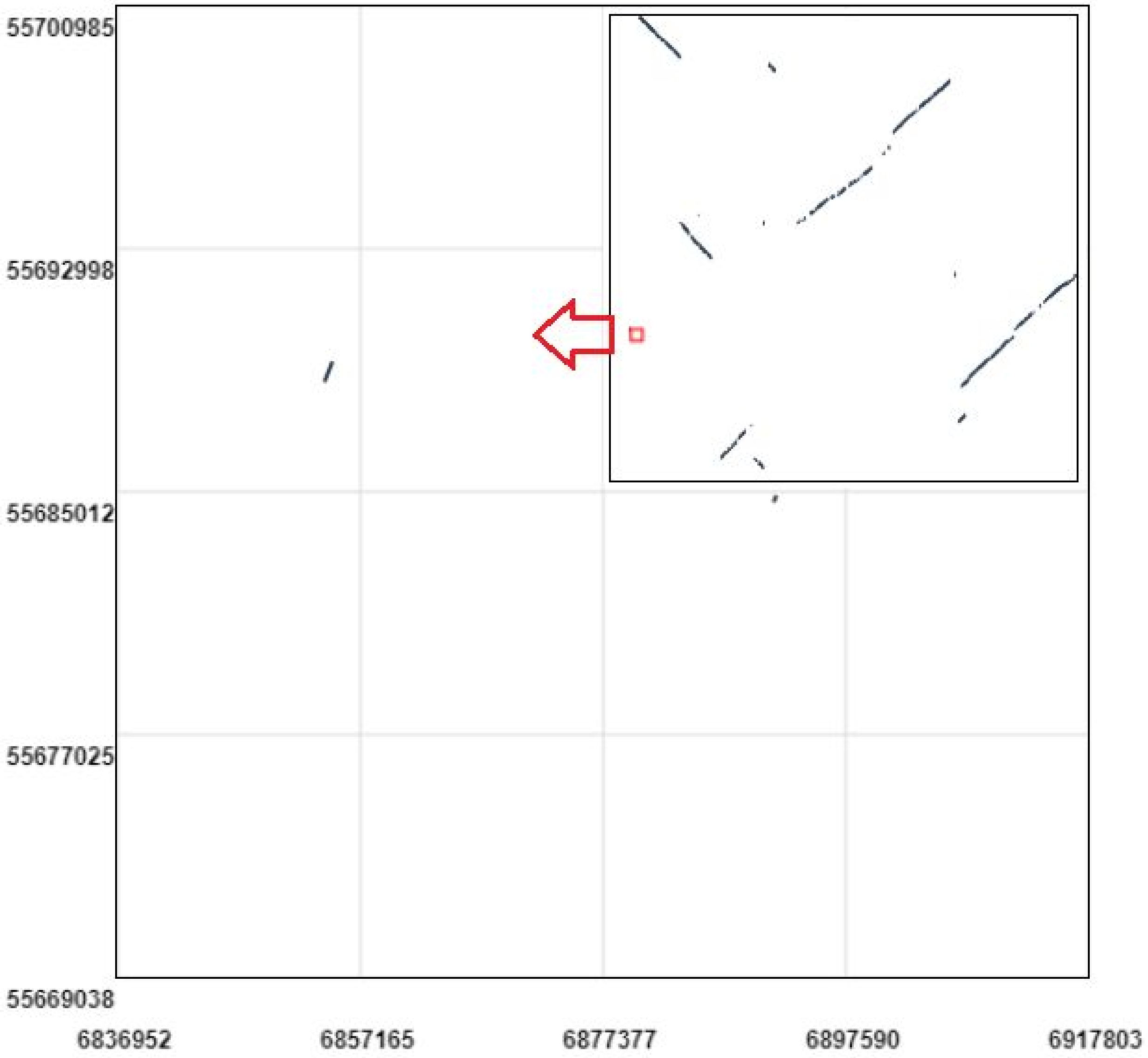

Figure 15. Zoomed region for blocks in part 1. Minimap is shown due to the amount of zoom performed.

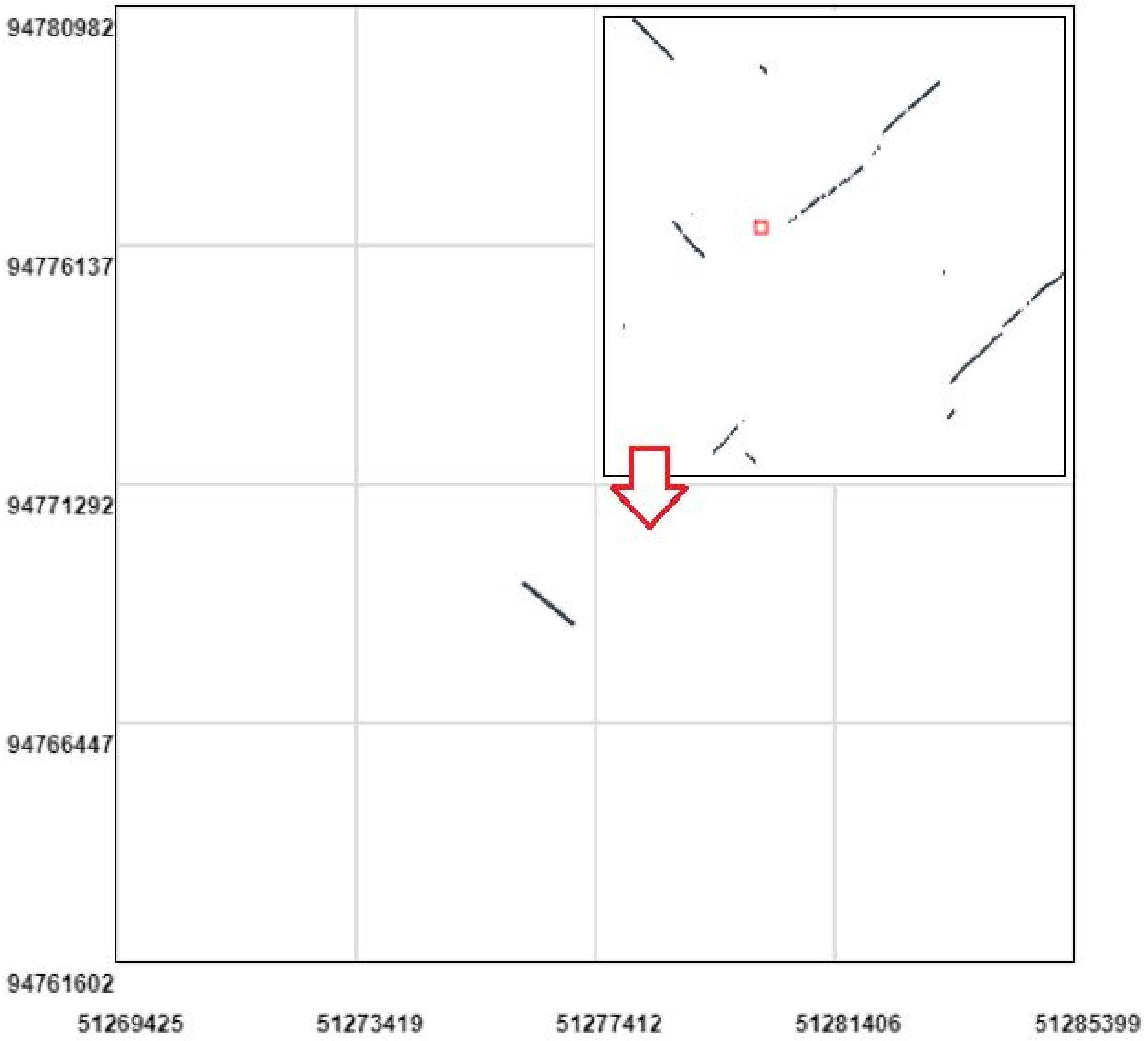

Figure 16. Zoomed region for the right block in part 2. Minimap is shown due to the amount of zoom performed.

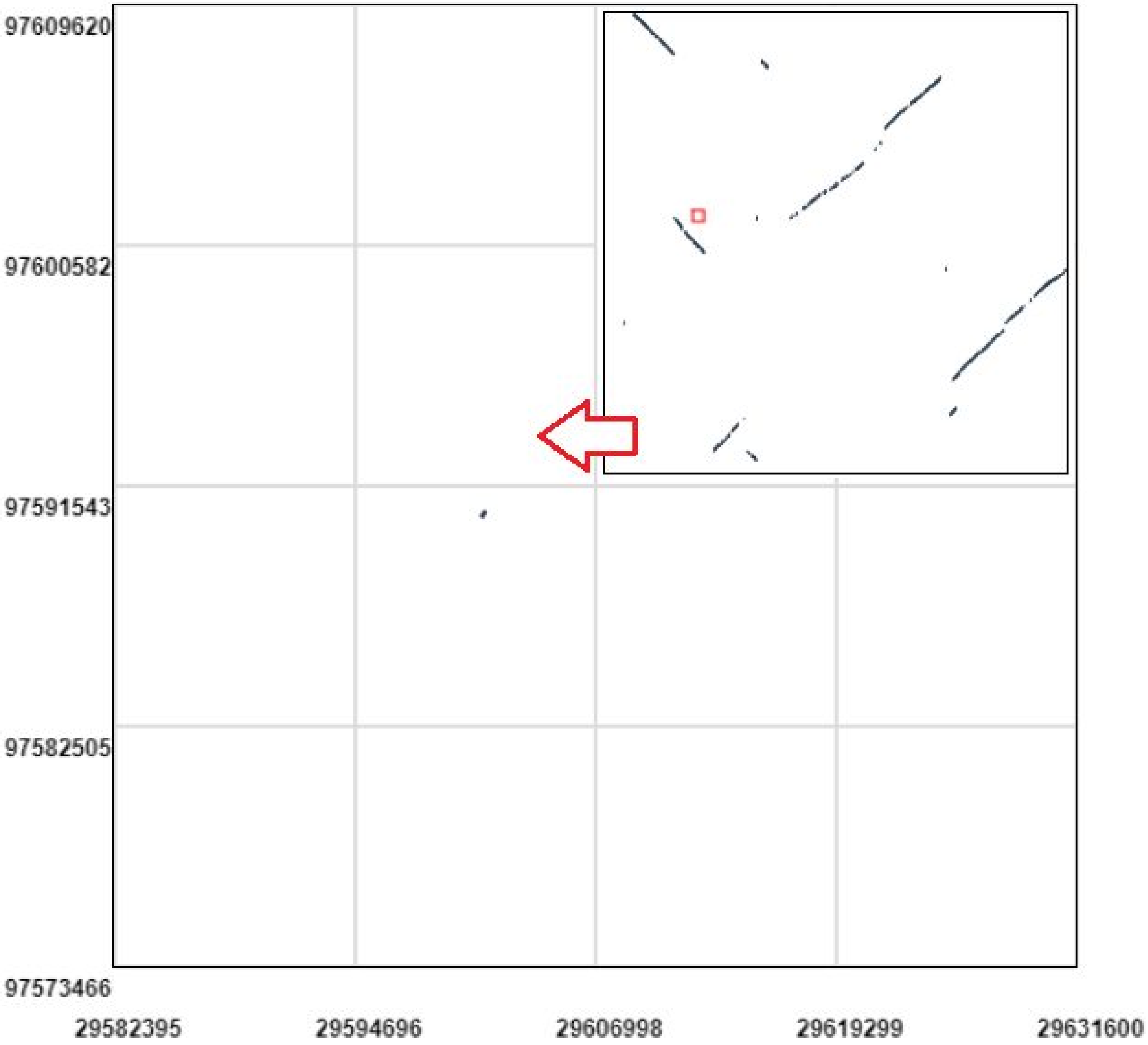

Figure 17. Zoomed region for the left block in part 2. Minimap is shown due to the amount of zoom performed.

NUCMER comparison

The NUCMER comparison of A1-T1 (*Aegilops tauschii* assem. chrom. 1 vs. *Triticum aestivum* pseudo-chrom. 1) produced a delta file of 455 MB. Running GNUPLOT with such file was not feasible, as it would crash. To obtain the picture representing the dotplot comparison between A1-T1, manual filtering was needed, which included:

- The resulting “fplot” and “rplot” files had to be shortened, by taking only the hits with 100% similarity. The following UNIX command was used for such purpose:

```
tail -n +2 file.[f/r]plot | awk '{if($3 > 99) print $0}' > file-100.[f/r]plot
```

- The script to plot both files had to be modified and is shown below (script is aimed at using X11 forwarding in the terminal):

```
set terminal x11 font "Courier,8"
set size 1,1
set grid
unset key
set border 15
set tics scale 0
set xlabel "CM008423.1_AEGILOPS_TAUSCHII"
set ylabel "PseudoChromosome1_TRITICUM_AESTIVUS"
set format "%.0f"
set xrange [1:492061028]
set yrange [1:524440854]
set style line 1  lt 1 lw 1 pt 1 ps 1
set style line 2  lt 1 lw 2 pt 6 ps 1
set style line 3  lt 1 lw 2 pt 6 ps 1
plot \
 "out-100.fplot" title "FWD" ls 1, \
 "out-100.rplot" title "REV" ls 1

pause -1
```

Pipelined execution

Using the script /bin/guidefastas.sh from the branch "inmemory_guided_chrom" available at GECKO's github (https://github.com/estebanpw/gecko/tree/inmemory_guided_chrom) it is possible to run full comparisons using the information generated by a previsualization comparison with CHROMEISTER.

All files were produced with parameters dimension: 1000, length: 200, similarity: 40 and word size: 32" (unless specified otherwise) and are available here[2]. See Figure 18 to 24 for the results.

[2] http://mango.ac.uma.es/compartir/csv

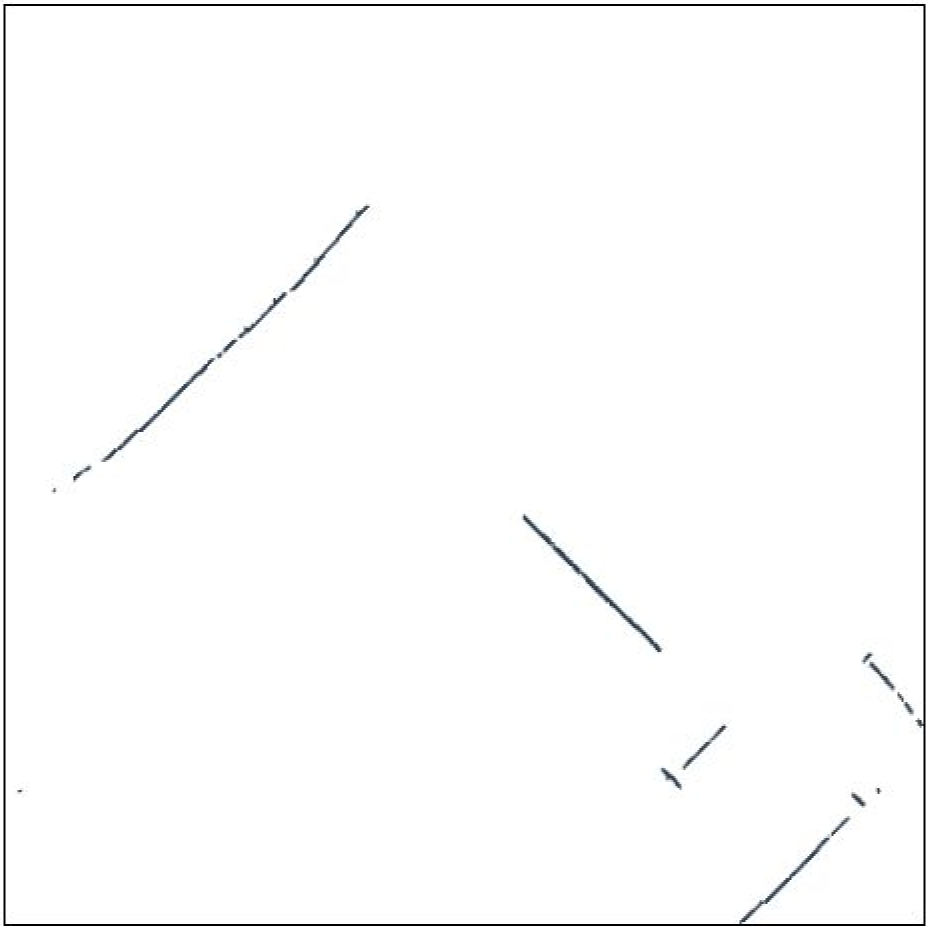

Figure 18. Horizontal view and dot-plot from the GECKO-MGV visualization of the B19-C9 comparison produced by GECKO with the information generated by CHROMEISTER.

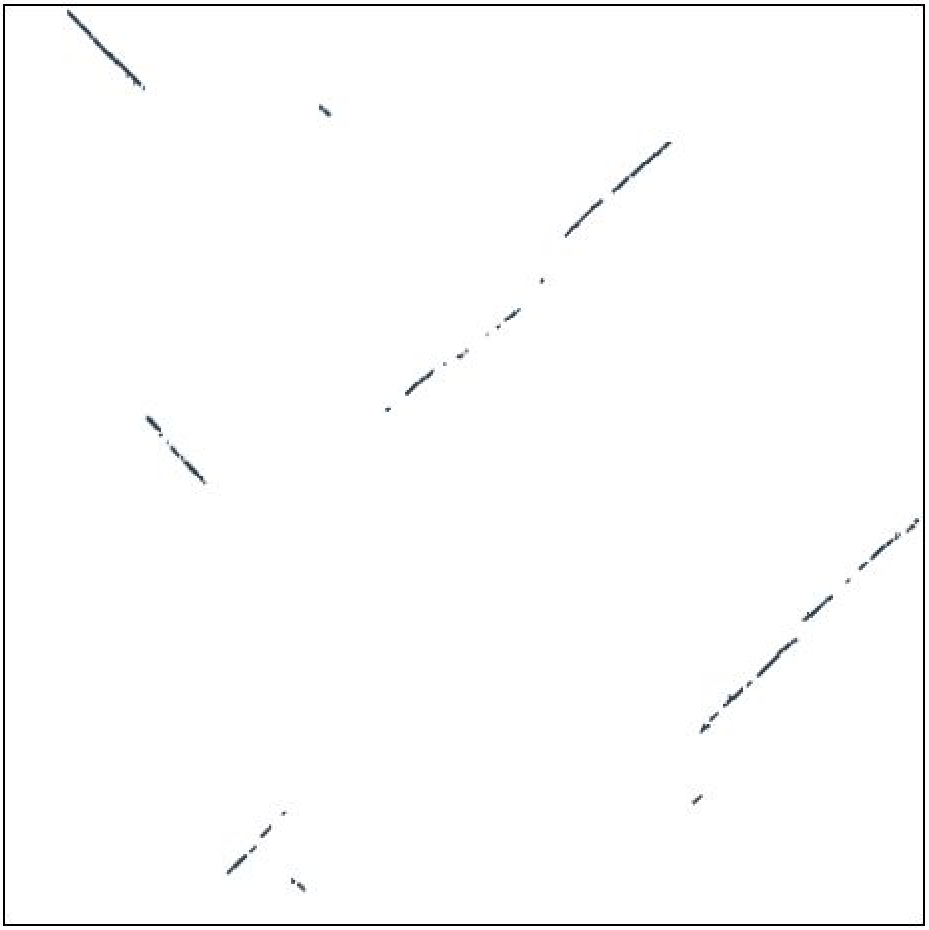

Figure 19. Horizontal view and dot-plot from the GECKO-MGV visualization of the HX-MX comparison produced by GECKO with the information generated by CHROMEISTER.

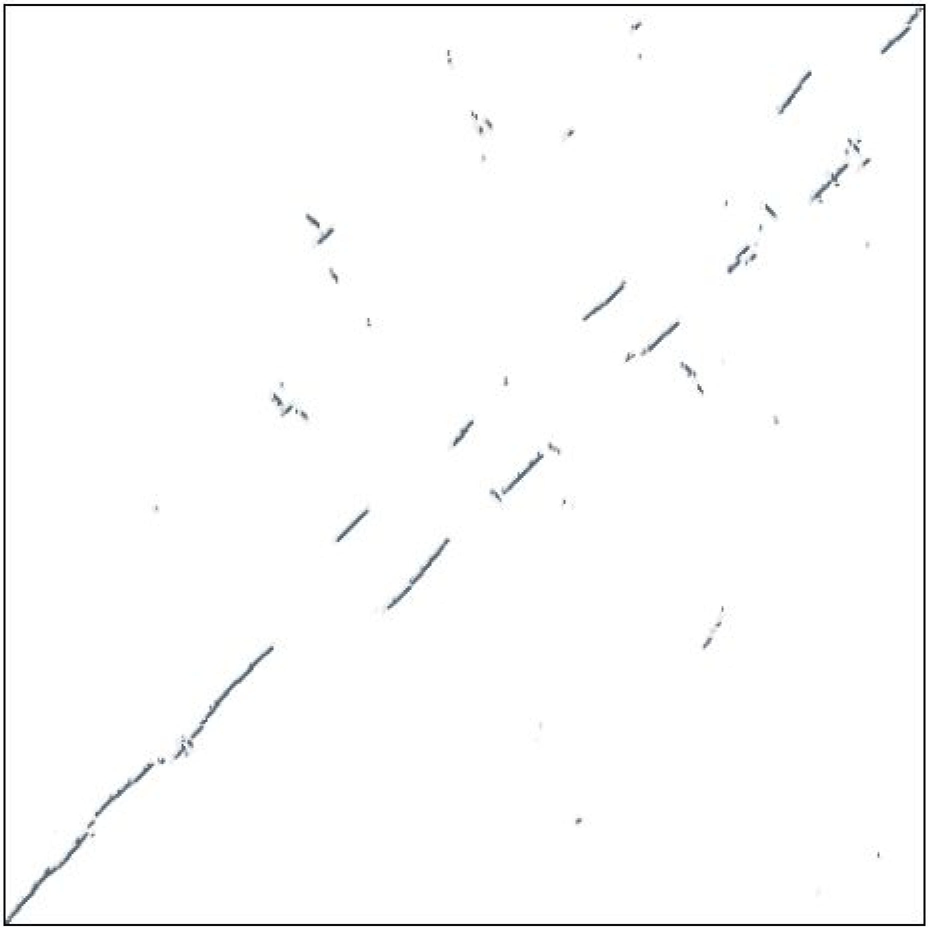

Figure 20. Horizontal view and dot-plot from the GECKO-MGV visualization of the BC-BC comparison produced by GECKO with the information generated by CHROMEISTER.

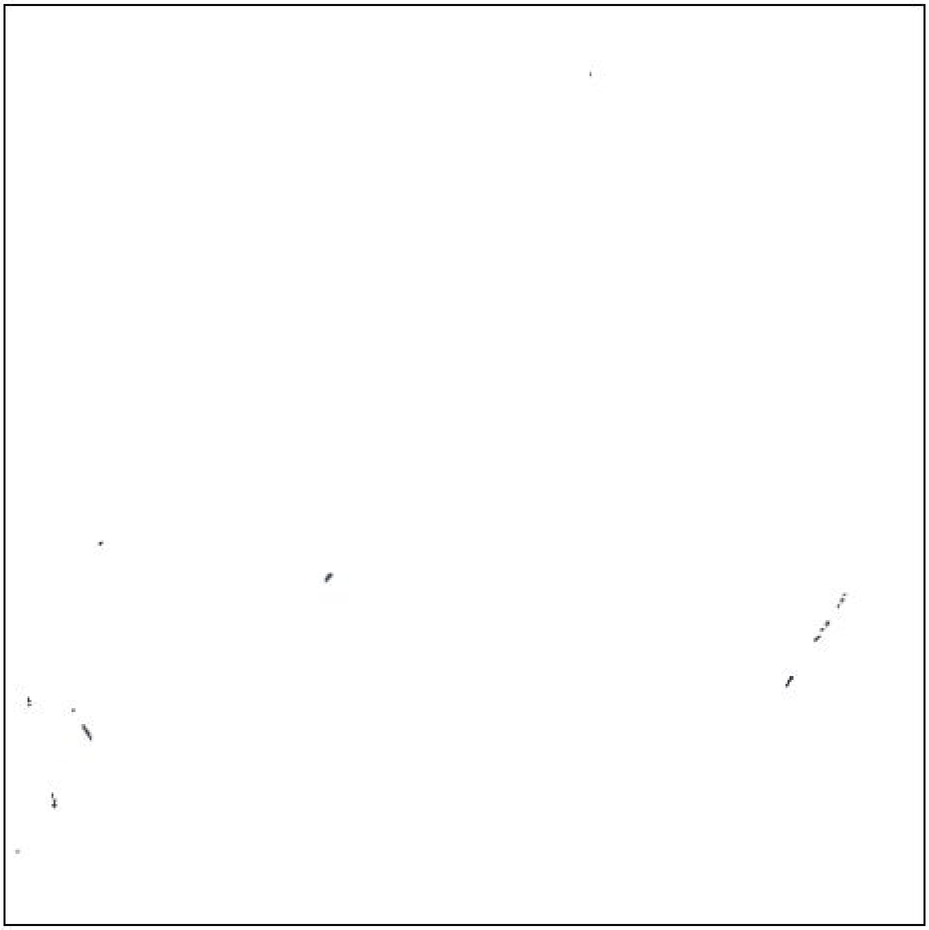

Figure 21. Horizontal view and dot-plot from the GECKO-MGV visualization of the M2-O1 comparison produced by GECKO with the information generated by CHROMEISTER. This one was filtered at only 200 bp of minimal length, since similarities were scarce.

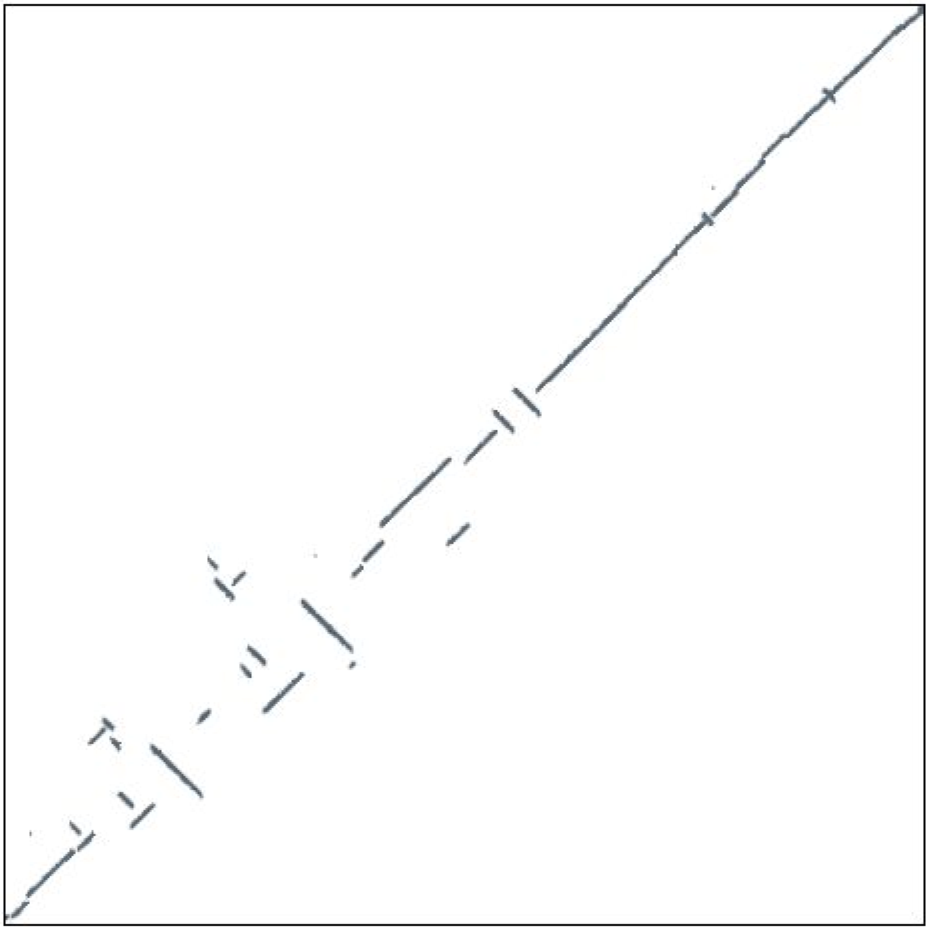

Figure 22. Horizontal view and dot-plot from the GECKO-MGV visualization of the A1-T1 comparison produced by GECKO with the information generated by CHROMEISTER. Filtered at 2,000 bp due to excess of fragments.

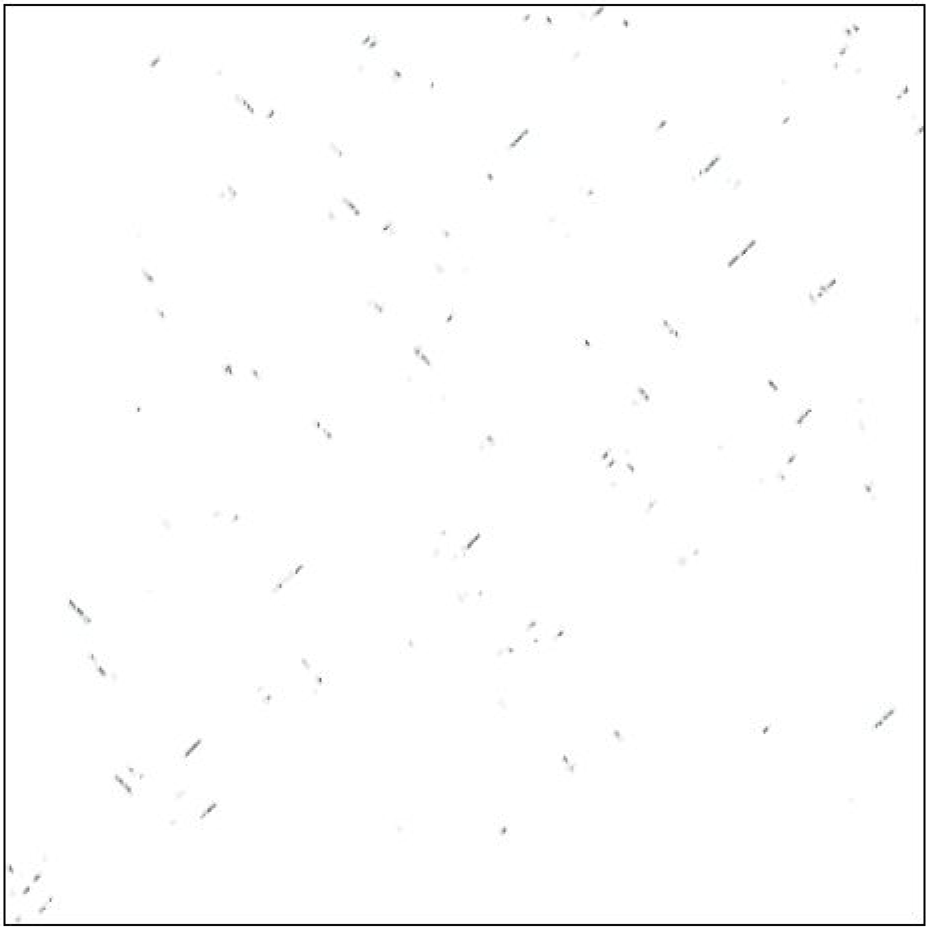

Figure 23. Horizontal view and dot-plot from the GECKO-MGV visualization of the HC-MC comparison produced by GECKO with the information generated by CHROMEISTER.

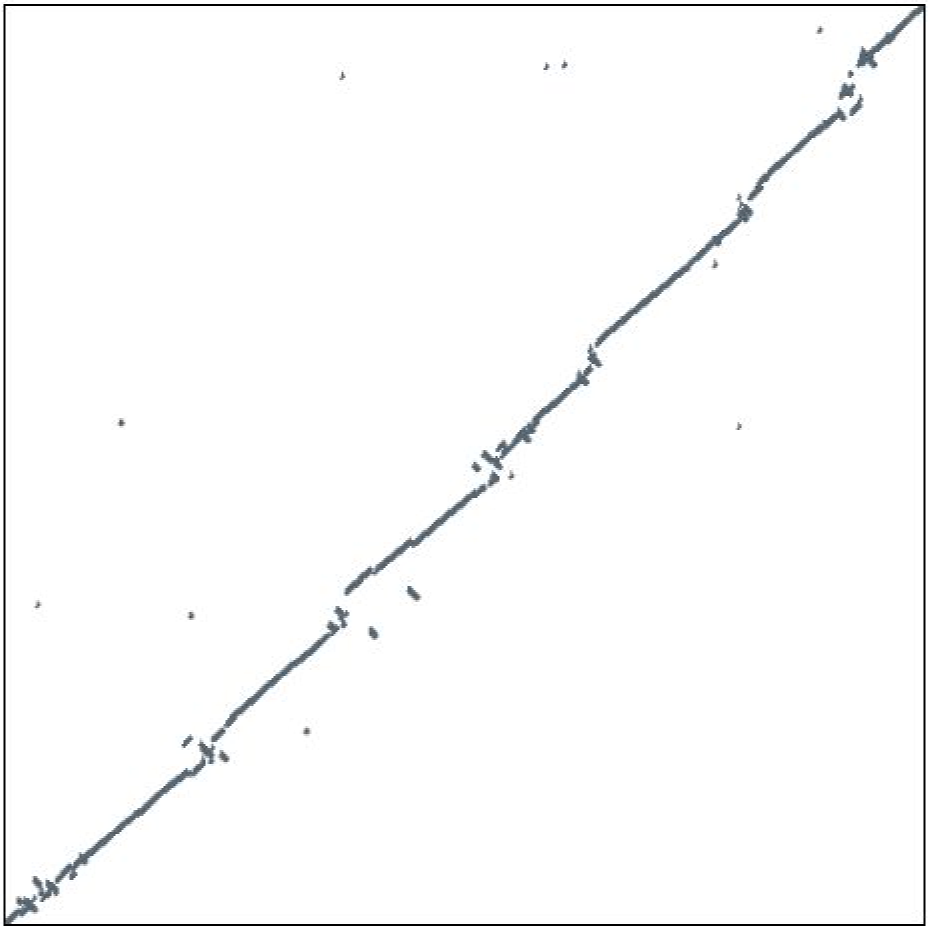

Figure 24. Horizontal view and dot-plot from the GECKO-MGV visualization of the AC-TC comparison produced by GECKO with the information generated by CHROMEISTER. Filtered at 2,000 bp due to excess of fragments.

Random perfect hits

This section addresses why the distribution of random hits can be ignored in the context of genomic comparisons.

The probability of a DNA sequence occurring somewhere else in another sequence is:

$$P(\text{sequence is matched}) = 1 - P(\text{sequence is not matched})$$

Therefore:

$$P(\text{sequence is not matched at position } i) = 1 - P(\text{sequence is matched at position } i)$$
$$P(\text{sequence is not matched at position } i) = 1 - \frac{1}{4^k}$$

Now we check that the sequence does not occur at position 1, 2, 3 … to N (being N the length of the sequence to be matched).

$$P(sequence\ is\ not\ matched\ anywhere) = (1 - \frac{1}{4^k})^N$$

And then we add that we have to check for a whole query sequence of length M:

$$P(no\ sequence\ from\ query\ is\ matched\ randomly) = ((1 - \frac{1}{4^k})^N)^M$$

Additional Table 1

Comparison name	Search space (bp^2)	Sequence names	Sequence lengths (bp)
B19-C9	$4.03 * 10^{15}$	*Bos taurus* Chr 19	$65 * 10^6$
		Canis familiaris Chr 9	$62 * 10^6$
HX-MX	$2.71 * 10^{16}$	*Homo sapiens* Chr X	$157 * 10^6$
		Mus musculus Chr X	$173 * 10^6$
BC-BC†	$1.18 * 10^{17}$	*Brassica oleracea* complete genome	$454 * 10^6$
		Brassica rapa complete genome	$260 * 10^6$
M2-O1	$1.54 * 10^{17}$	*Monodelphis domestica* Chr 2	$550 * 10^6$
		Ovis aries Chr 1	$280 * 10^6$
A1-T1	$2.57 * 10^{17}$	*Aegilops tauschii* assem. Chr 1	$492 * 10^6$
		Triticum aestivum pseudo-Chr 1	$524 * 10^6$
HC-MC†	$8.69 * 10^{18}$	*Homo sapiens* complete genome**	$314 * 10^7$
		Mus musculus complete genome**	$277 * 10^7$
AC-TC†	$1.39 * 10^{19}$	*Aegilops tauschii* complete genome**	$329 * 10^7$
		Triticum aestivum complete genome**	$423 * 10^7$

Additional Table 1. Sequences involved in the experiments depicted in the Supplementary Material. The assigned comparison name is composed as the first letter of the genome from where the sequence was taken plus the chromosome number (if any) or the letter “C” for short of “Complete”, if the whole genome was compared. (*) Only the D genome components were used from this hexaploid organism. A double asterisk ‘**’ implies that the sequence is the complete genome but not the assembly, i.e. all chromosomes sorted by their lengths. (†) The

sequence represents the complete genome by building one file composed of all chromosomes in sequential order by size.

Additional Table 2

Comparison name	Search space (bp^2)	Sequence names	Sequence lengths (bp)
B19-C9	$4.03 * 10^{15}$	*Bos taurus* chr. 19	$65 * 10^{6}$
		Canis familiaris chr. 9	$62 * 10^{6}$
HX-MX	$2.07 * 10^{16}$	*Homo sapiens* chr. X	$157 * 10^{6}$
		Mus musculus chr. X	$173 * 10^{6}$
BC-BC	$1.18 * 10^{17}$	*Brassica oleracea* complete genome	$454 * 10^{6}$
		Brassica rapa complete genome	$260 * 10^{6}$
M2-O1	$1.54 * 10^{17}$	*Monodelphis domestica* chr. 2	$550 * 10^{6}$
		Ovis aries chr. 1	$280 * 10^{6}$
MAC2-GO2	$2.04 * 10^{17}$	*Macaca mulatta* chr. 1 and 2	$424 * 10^{6}$
		Gorilla gorilla chr. 1 and 2	$480 * 10^{6}$
A1-T1	$2.57 * 10^{17}$	*Aegilops tauschii* assem. chr. 1	$492 * 10^{6}$
		Triticum aestivum pseudo-chr. 1	$524 * 10^{6}$
GC-EC	$2.52 * 10^{18}$	*Gallus gallus* complete genome	$104 * 10^{7}$
		Equus caballus complete genome	$240 * 10^{7}$
HC-MC	$8.69 * 10^{18}$	*Homo sapiens* complete genome	$314 * 10^{7}$
		Mus musculus complete genome	$277 * 10^{7}$
AC-TC	$1.39 * 10^{19}$	*Aegilops tauschii* complete genome[3]	$329 * 10^{7}$
		Triticum aestivum complete genome[2]	$423 * 10^{7}$

Additional Table 2 Sequences involved in the manuscript or supplementary material.

[3] As built from from the chromosomes obtained from the previously specified resource.

Additional Table 3

		CGALN		NUCMER		CHROMEISTER	
Dataset	Search Sp. (bp^2)	Time (m)	RAM (MB)	Time (m/adjusted)	RAM (MB)	Time (m)	RAM (MB)
B19-C9	$4.03 * 10^{15}$	1.63	755	1.49/2.13	812	**0.89**	**994**
HX-MX	$2.07 * 10^{16}$	5.93	2,032	3.60/5.14	2,221	**1.29**	**998**
BC-BC	$1.18 * 10^{17}$	141.26	583	8.90/13.61	5,814	**2.42**	**1,022**
M2-O1	$1.54 * 10^{17}$	17.04	3,618	9.18/15.14	6,436	**2.84**	**1,017**
A1-T1	$2.57 * 10^{17}$	DNF	DNF	7.50/11.40	8,357	**2.84**	**1,022**
HC-MC	$8.69 * 10^{18}$	DNF	DNF	136.18/168.86	46,091	**13.77**	**1,022**
AC-TC	$1.39 * 10^{19}$	DNF	DNF	121.10/176.80	52,021	**15.96**	**1,022**

Additional Table 3. Comparison of the best performance of either CGALN, NUCMER and CHROMEISTER. The datasets are sorted by search space size. From left to right column: the sequences compared, search space (in base pairs to the power of two), time (in minutes) and memory consumption (megabytes) for each method. The acronym "DNF" stands for "Did Not Finish", which was set after 100 hours of computing time. The time column of NUCMER indicates that the program used more than one CPU and therefore the adjusted time is shown.

Coverage comparison using MEGABLAST

The parameters employed for the MEGABLAST comparison of coverage were:

blastn -task megablast -query <query> -db <db> -out <out> -word_size 32 -ungapped

The other software employed was used with parameters as described previously.

Distance aggregation

In the case of using the proposed method for evolutionary studies, it might be worthy to discuss the aggregation of individual scores in case that comparisons were performed at chromosome or subsequence levels. Notice that for a genome composed of several chromosomes, the score must be aggregated in order to generate a single metric (i.e. a metric that represents the score of the n chromosomes of one genome compared to the n' chromosomes of another genome). A trivial procedure to do so is to concatenate the chromosomes into a larger genome and then perform the comparison; however, such a scenario is not always feasible. For the cases where data is split, determining an aggregation metric is no trivial task and should incorporate evolutionary models. However, we propose a simple aggregation model. Take $d_{i,j}^{a,b}$ as the

distance between chromosomes i and j of genomes a and b. Then, the scoring distance between chromosome i and genome b (notice that chromosome i could have split in several chromosomes in genome b) is given by Algorithm 1:

Algorithm 1 Scoring chromosome c_x^a against chromosomes $C^b = \{c_1^b, c_2^b, ..., c_i^b, ..., c_n^b\}$

procedure SCORE_CHROMOSOME($Scores\ D^{c_x^a, C^b}$, $Float\ threshold$)
  s ← sort_ascending($D^{c_x^a, C^b}$)
  score ← vector()
  current ← first(s)
  append($current$, $score$)
  current ← next($current$, s)
  next ← next($current$, s)
  while $next - current > threshold$ **do**
    append($current$, $score$)
    current ← next
    next ← next($current$, s)
  end while
  return $\frac{sum(score)}{\lambda(size(score))}$
end procedure

Algorithm 1. Procedure to score a single sequence compared to several others.

The algorithm greedily incorporates scores from split chromosomes while significant homologies are found (as determined by the threshold). Once no more significant homologies are found, the sum of the used scores is normalized by $\lambda(x)$. Such function should penalize the number of different sequences where homologies were found by using an evolutionary model. For instance, a Poisson distribution with the average number of splits per compared chromosome as parameter (see section “Poisson distribution in split number of chromosomes”).

Poisson distribution in split number of chromosomes

By performing a poisson fit on the number of times the chromosomes are similar to other chromosomes (from both their and other species) (i.e. how many times a chromosome split in others), a fairly well fit is obtained to a Poisson distribution. Figure 24 depicts the fit produced by the greedy algorithm using a threshold $t = 0.1$, and successive Figures 25 and 26 for $t = 0.2$ and $t = 0.3$, respectively.

The experiment comprised the comparison of all versus all chromosomes for every pair of genomes, which is summarized in Table 17.

Genome	Number of chromosomes
Bos taurus	31

Canis familiaris	40
Equus caballus	33
Gallus gallus	31
Gorilla gorilla	25
Homo sapiens	25
Macaca mulatta	22
Mus musculus	22
Pan troglodytes	26
Pongo abelii	26
Callithrix jacchus	24

Table 17. Dataset used to empirically determine the nature of the distribution of split homologies in chromosomes.

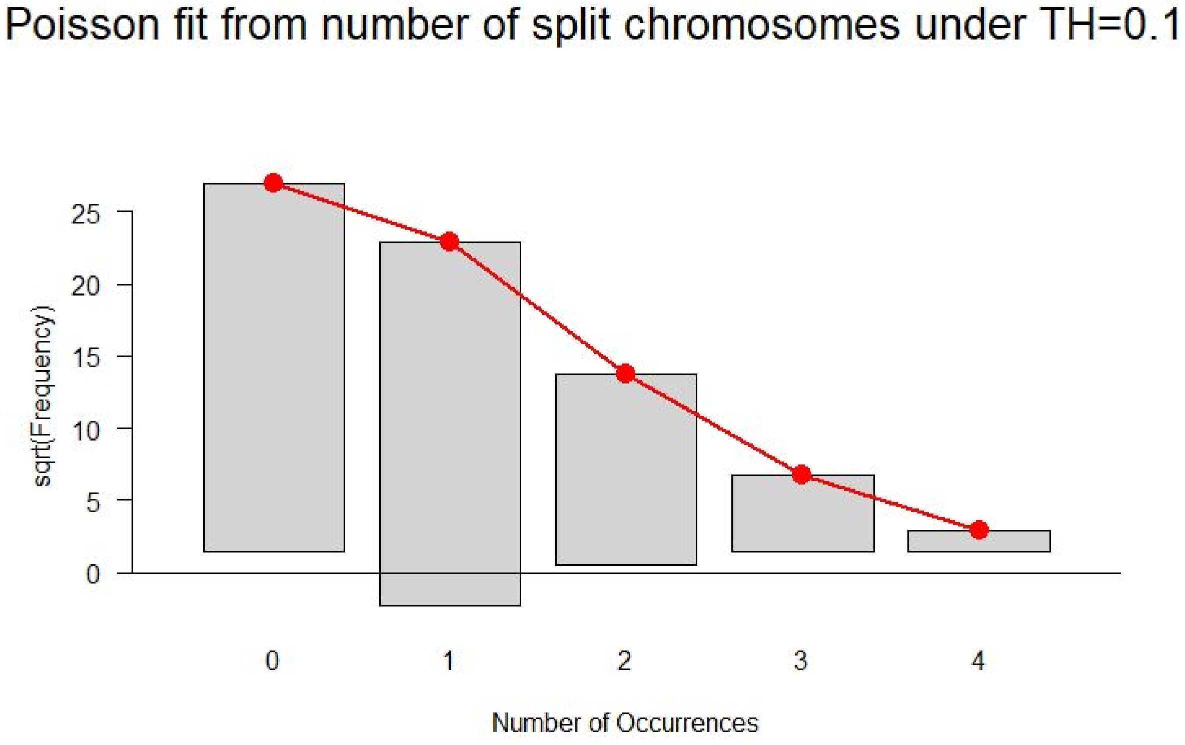

Figure 25. Poisson fit to the number of homology splits in chromosomes for $t = 0.1$. The x-axis represents how many times each chromosome is split, meaning that "0" represents that the homology of a given chromosome was either found in just one other chromosome or was not found at all. On the other hand, a value of "1" or more indicates that the chromosome's homology was split more than once.

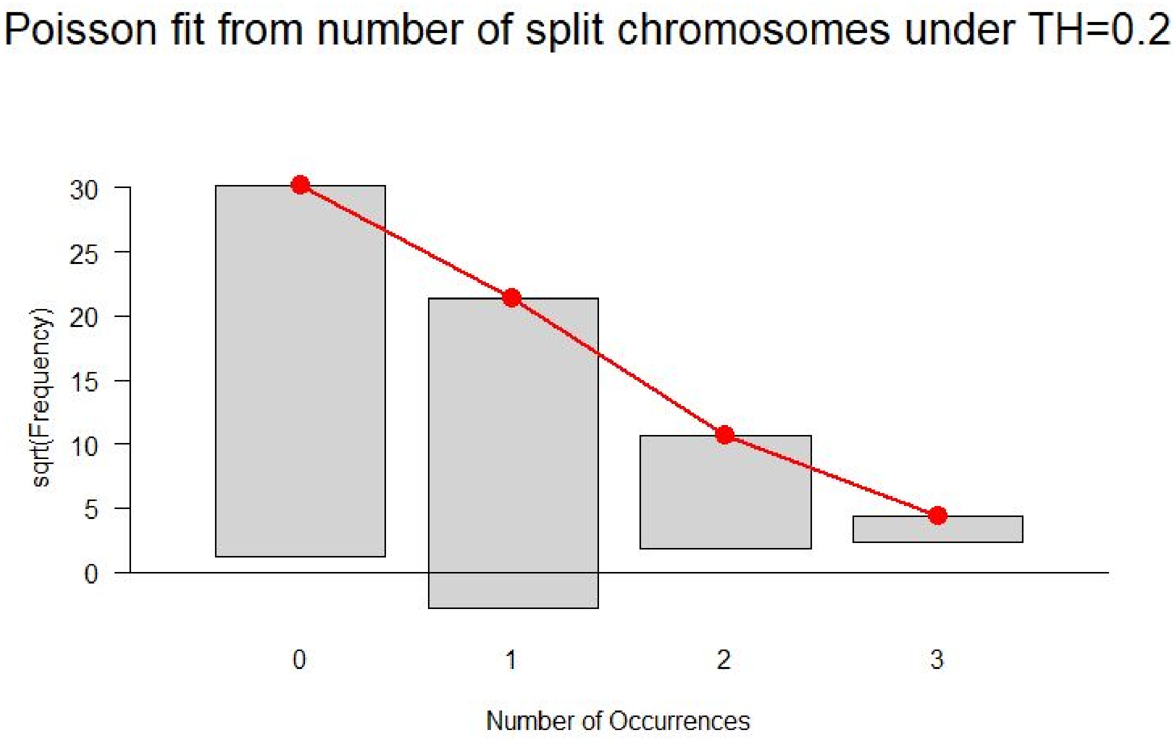

Figure 26. Poisson fit to the number of homology splits in chromosomes for $t = 0.2$. The x-axis represents how many times each chromosome is split, meaning that “0” represents that the homology of a given chromosome was either found in just one other chromosome or was not found at all. On the other hand, a value of “1” or more indicates that the chromosome’s homology was split more than once.

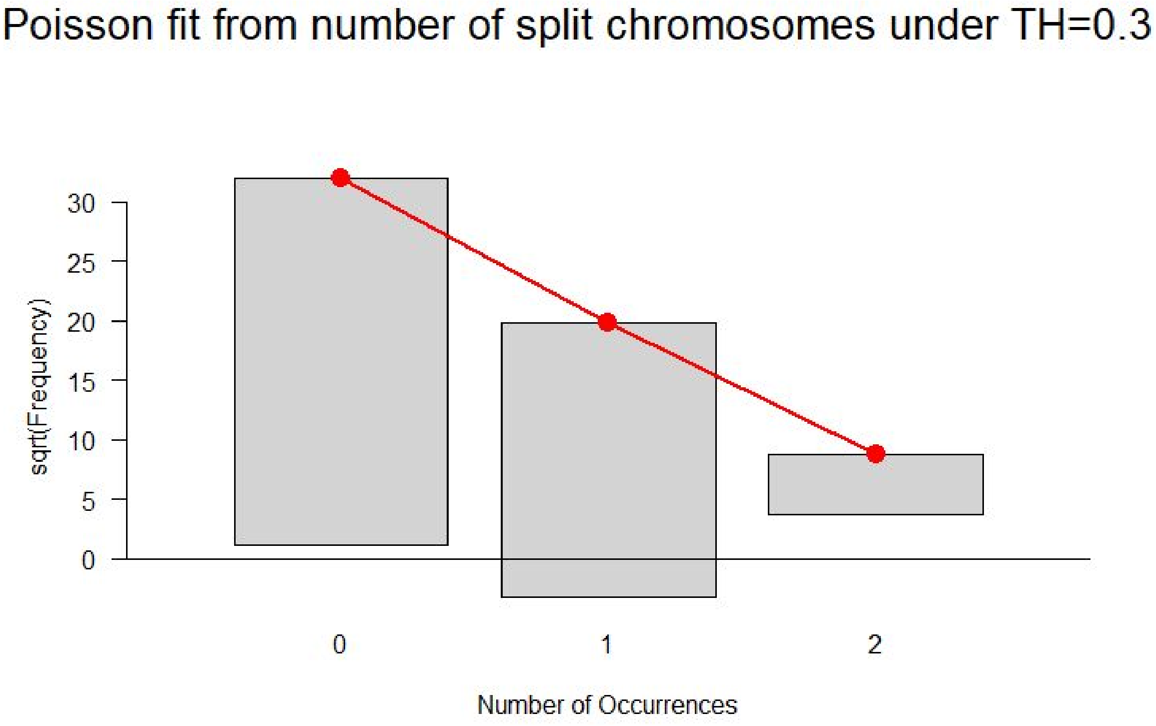

Figure 27. Poisson fit to the number of homology splits in chromosomes for $t = 0.3$. The x-axis represents how many times each chromosome is split, meaning that "0" represents that the homology of a given chromosome was either found in just one other chromosome or was not found at all. On the other hand, a value of "1" or more indicates that the chromosome's homology was split more than once.

List of copyright permissions for the pictures shown in Figure 3

1. *Vitruvian Man*
   Vitruvian Man (Homo cuadratus). Pixabay license.
   Figure was partially cropped.
   https://pixabay.com/illustrations/leonardo-da-vinci-vitruvian-man-1125056/
   Pixabay license: https://pixabay.com/service/license/
2. *Pan troglodytes*
   Pan troglodytes (Blumenbach, 1775). Male Chimpanzee at La Vallée de Singes, at Romagne (Vienne, Poitou-Charentes), France. CC BY-SA 3.0.
   Figure was partially cropped.
   https://commons.wikimedia.org/wiki/File:Pan_troglodytes_(male).jpg
   CC BY-SA 3.0: https://creativecommons.org/licenses/by-sa/3.0/
3. *Pan paniscus*
   Male Bonobo in Apenheul Primate Park, Apeldoorn, NL. Natataek at English Wikivoyage. CC BY-SA 3.0.
   Figure was partially cropped.
   https://commons.wikimedia.org/wiki/File:Apeldoorn_Apenheul_zoo_Bonobo.jpg
   CC BY-SA 3.0: https://creativecommons.org/licenses/by-sa/3.0/
4. *Gorilla gorilla*
   Gorilla gorilla au zoo de Melbourne (Australie). Macinate - CC BY 2.0.
   Figure was partially cropped.
   https://es.wikipedia.org/wiki/Archivo:Gorille.jpg
   CC BY 2.0: https://creativecommons.org/licenses/by/2.0/deed.en
5. *Pongo abelii*
   Nahaufnahme des männlichen Sumatra-Orang-Utans Bimbo im Pongoland des Zoos Leipzig. Fiver, der Hellseher - Own work. CC BY-SA 4.0.
   Figure was partially cropped.
   https://commons.wikimedia.org/wiki/File:Sumatra-Orang-Utan_im_Pongoland.jpg
   CC BY-SA 4.0: https://creativecommons.org/licenses/by-sa/4.0/legalcode
6. *Nomascus leucogenys*
   White-cheeked Crested Gibbon, Nomascus leucogenys, at the Lincoln Park Zoo in Chicago. Taken with a Canon Digital Rebel and a Canon 70-300/4-5.6 IS USM. ISO 800, 1/13s. Grendelkhan - Own work. CC BY-SA 3.0.
   Figure was partially cropped.
   https://fr.wikipedia.org/wiki/Fichier:Nomascus_leucogenys.gk.jpg
   CC BY-SA 3.0: https://creativecommons.org/licenses/by-sa/3.0/

7. *Papio anubis*
   An Olive baboon (Papio anubis) in the Ngorongoro Conservation Area in Tanzania. Muhammad Mahdi Karim - Own work. GFDL 1.2.
   Figure was partially cropped.
   https://en.wikipedia.org/wiki/File:Olive_baboon_Ngorongoro.jpg
   GFDL 1.2 https://www.gnu.org/licenses/old-licenses/fdl-1.2.en.html
8. *Macaca mulatta*
   Rhesus macaque (Macaca mulatta mulatta) female, Satpura National Park, MP, India. Charles J Sharp - Own work. CC BY-SA 4.0.
   Figure was partially cropped.
   https://commons.wikimedia.org/wiki/File:Rhesus_macaque_(Macaca_mulatta_mulatta)_female.jpg
   CC BY-SA 4.0: https://creativecommons.org/licenses/by-sa/4.0/legalcode
9. *Macaca fascicularis*
   Macaca fascicularis at Ngarai Sianok, Bukittinggi, West Sumatra. Sakurai Midori - Own work. CC BY-SA 3.0.
   Figure was partially cropped.
   https://commons.wikimedia.org/wiki/File:Ngarai_Sianok_sumatran_monkey.jpg
   CC BY-SA 3.0: https://creativecommons.org/licenses/by-sa/3.0/
10. *Chlorocebus sabaeus*
    Gambia06Bijilo0015. tjabeljan - Gambia06Bijilo0015. CC BY 2.0.
    Figure was partially cropped.
    https://commons.wikimedia.org/wiki/File:Gambia06Bijilo0015_(5421078756).jpg
    CC BY 2.0: https://creativecommons.org/licenses/by/2.0/deed.en
11. *Callithrix jacchus*
    Common Marmoset (Callithrix jacchus) at Aquazoo-Löbbecke-Museum Düsseldorf. © Raimond Spekking / CC BY-SA 4.0 (via Wikimedia Commons). CC BY-SA 4.0.
    Figure was partially cropped.
    https://commons.wikimedia.org/wiki/File:Wei%C3%9Fb%C3%BCschelaffe_(Callithrix_jacchus).jpg
    CC BY-SA 4.0: https://creativecommons.org/licenses/by-sa/4.0/legalcode
12. *Microcebus murinus*
    A large-eyed lemur perched on a wooden rod with the long furry tail dropping down. photographer: Gabriella Skollar; editor: Rebecca Lewis - Own work. CC BY-SA 3.0.
    Figure was partially cropped.
    https://commons.wikimedia.org/wiki/File:Gray_Mouse_Lemur_1.JPG
    CC BY-SA 3.0: https://creativecommons.org/licenses/by-sa/3.0/

References

1. Elena, Santiago F., Thomas S. Whittam, Cynthia L. Winkworth, Margaret A. Riley, and Richard E. Lenski. "Genomic divergence of Escherichia coli strains: evidence for

horizontal transfer and variation in mutation rates." International Microbiology 8, no. 4 (2005): 271-278.
