# Supplementary material for: Ultra-fast genome comparison for large-scale genomic experiments
Source: Sci Rep. 2019 Jul 16;9:10274. doi: 10.1038/s41598-019-46773-w (PMC6635410; doi:10.1038/s41598-019-46773-w)
Supplement: Supplementary file 2 — Additional File 1 [file 41598_2019_46773_MOESM2_ESM.pdf]

Ultra-fast genome comparison for large-scale genomic experiments

Additional File 1

Esteban Pérez-Wohlfeil[1], Sergio Diaz-del-Pino[1] and Oswaldo Trelles[1]

1. Computer Architecture Department, University of Málaga - Instituto de Investigación Biomédica de Málaga-IBIMA, Spain

List of copyright permissions for the pictures shown in Figure 3

1. *Vitruvian Man*
   Vitruvian Man (Homo cuadratus). Pixabay license.
   Figure was partially cropped.
   https://pixabay.com/illustrations/leonardo-da-vinci-vitruvian-man-1125056/
   Pixabay license: https://pixabay.com/service/license/
2. *Pan troglodytes*
   Pan troglodytes (Blumenbach, 1775). Male Chimpanzee at La Vallée de Singes, at Romagne (Vienne, Poitou-Charentes), France. CC BY-SA 3.0.
   Figure was partially cropped.
   https://commons.wikimedia.org/wiki/File:Pan_troglodytes_(male).jpg
   CC BY-SA 3.0: https://creativecommons.org/licenses/by-sa/3.0/
3. *Pan paniscus*
   Male Bonobo in Apenheul Primate Park, Apeldoorn, NL. Natataek at English Wikivoyage. CC BY-SA 3.0.
   Figure was partially cropped.
   https://commons.wikimedia.org/wiki/File:Apeldoorn_Apenheul_zoo_Bonobo.jpg
   CC BY-SA 3.0: https://creativecommons.org/licenses/by-sa/3.0/
4. *Gorilla gorilla*
   Gorilla gorilla au zoo de Melbourne (Australie). Macinate - CC BY 2.0.
   Figure was partially cropped.
   https://es.wikipedia.org/wiki/Archivo:Gorille.jpg
   CC BY 2.0: https://creativecommons.org/licenses/by/2.0/deed.en
5. *Pongo abelii*
   Nahaufnahme des männlichen Sumatra-Orang-Utans Bimbo im Pongoland des Zoos Leipzig. Fiver, der Hellseher - Own work. CC BY-SA 4.0.
   Figure was partially cropped.
   https://commons.wikimedia.org/wiki/File:Sumatra-Orang-Utan_im_Pongoland.jpg
   CC BY-SA 4.0: https://creativecommons.org/licenses/by-sa/4.0/legalcode
6. *Nomascus leucogenys*
   White-cheeked Crested Gibbon, Nomascus leucogenys, at the Lincoln Park Zoo in Chicago. Taken with a Canon Digital Rebel and a Canon 70-300/4-5.6 IS USM. ISO 800, 1/13s. Grendelkhan - Own work. CC BY-SA 3.0.

Figure was partially cropped.
https://fr.wikipedia.org/wiki/Fichier:Nomascus_leucogenys.gk.jpg
CC BY-SA 3.0: https://creativecommons.org/licenses/by-sa/3.0/

7. *Papio anubis*
   An Olive baboon (Papio anubis) in the Ngorongoro Conservation Area in Tanzania. Muhammad Mahdi Karim - Own work. GFDL 1.2.
   Figure was partially cropped.
   https://en.wikipedia.org/wiki/File:Olive_baboon_Ngorongoro.jpg
   GFDL 1.2 https://www.gnu.org/licenses/old-licenses/fdl-1.2.en.html
8. *Macaca mulatta*
   Rhesus macaque (Macaca mulatta mulatta) female, Satpura National Park, MP, India. Charles J Sharp - Own work. CC BY-SA 4.0.
   Figure was partially cropped.
   https://commons.wikimedia.org/wiki/File:Rhesus_macaque_(Macaca_mulatta_mulatta)_female.jpg
   CC BY-SA 4.0: https://creativecommons.org/licenses/by-sa/4.0/legalcode
9. *Macaca fascicularis*
   Macaca fascicularis at Ngarai Sianok, Bukittinggi, West Sumatra. Sakurai Midori - Own work. CC BY-SA 3.0.
   Figure was partially cropped.
   https://commons.wikimedia.org/wiki/File:Ngarai_Sianok_sumatran_monkey.jpg
   CC BY-SA 3.0: https://creativecommons.org/licenses/by-sa/3.0/
10. *Chlorocebus sabaeus*
    Gambia06Bijilo0015. tjabeljan - Gambia06Bijilo0015. CC BY 2.0.
    Figure was partially cropped.
    https://commons.wikimedia.org/wiki/File:Gambia06Bijilo0015_(5421078756).jpg
    CC BY 2.0: https://creativecommons.org/licenses/by/2.0/deed.en
11. *Callithrix jacchus*
    Common Marmoset (Callithrix jacchus) at Aquazoo-Löbbecke-Museum Düsseldorf. © Raimond Spekking / CC BY-SA 4.0 (via Wikimedia Commons). CC BY-SA 4.0.
    Figure was partially cropped.
    https://commons.wikimedia.org/wiki/File:Wei%C3%9Fb%C3%BCschelaffe_(Callithrix_jacchus).jpg
    CC BY-SA 4.0: https://creativecommons.org/licenses/by-sa/4.0/legalcode
12. *Microcebus murinus*
    A large-eyed lemur perched on a wooden rod with the long furry tail dropping down. photographer: Gabriella Skollar; editor: Rebecca Lewis - Own work. CC BY-SA 3.0.
    Figure was partially cropped.
    https://commons.wikimedia.org/wiki/File:Gray_Mouse_Lemur_1.JPG
    CC BY-SA 3.0: https://creativecommons.org/licenses/by-sa/3.0/
